# SLC38A10 Regulate Glutamate Homeostasis and Modulate the AKT/TSC2/mTOR Pathway in Mouse Primary Cortex Cells

**DOI:** 10.3389/fcell.2022.854397

**Published:** 2022-04-05

**Authors:** Rekha Tripathi, Tanya Aggarwal, Frida A. Lindberg, Anna H. Klemm, Robert Fredriksson

**Affiliations:** ^1^ Department of Pharmaceutical Bioscience, Unit of Molecular Neuropharmacology, Uppsala University, Uppsala, Sweden; ^2^ BioImage Informatics Facility, SciLifeLab, Division of Visual Information and Interaction, Department of Information Technology, Uppsala, Sweden

**Keywords:** solute carriers transporter, primary cortex cultures, starvation, SLC38A10, mTOR

## Abstract

Glutamate acts as a critical regulator of neurotransmitter balance, recycling, synaptic function and homeostasis in the brain and glutamate transporters control glutamate levels in the brain. SLC38A10 is a member of the SLC38 family and regulates protein synthesis and cellular stress responses. Here, we uncover the role of SLC38A10 as a transceptor involved in glutamate-sensing signaling pathways that control both the glutamate homeostasis and mTOR-signaling. The culture of primary cortex cells from SLC38A10 knockout mice had increased intracellular glutamate. In addition, under nutrient starvation, KO cells had an impaired response in amino acid-dependent mTORC1 signaling. Combined studies from transcriptomics, protein arrays and metabolomics established that SLC38A10 is involved in mTOR signaling and that SLC38A10 deficient primary cortex cells have increased protein synthesis. Metabolomic data showed decreased cholesterol levels, changed fatty acid synthesis, and altered levels of fumaric acid, citrate, 2-oxoglutarate and succinate in the TCA cycle. These data suggests that SLC38A10 may act as a modulator of glutamate homeostasis, and mTOR-sensing and loss of this transceptor result in lower cholesterol, which could have implications in neurodegenerative diseases.

## Introduction

The Solute Carriers (SLCs) control fundamental metabolic and cellular functions in cells (Sundberg et al., 2008). These proteins play an essential role in monitoring the flow of a wide range of substances over the cell membrane, substances such as sugar, amino acids, nucleotides, and inorganic ions (Hediger et al., 2004). The human SLC family consists of over 400 annotated members, organized into 66 subfamilies and constitutes the second largest family of membrane proteins (Sundberg et al., 2008; Perland et al., 2017; Kandasamy et al., 2018). In a recent review, 287 SLCs had verified expression in the brain; moreover, there are 71 polymorphisms linked with human brain disorders such as epilepsy, neurodegenerative diseases and autism(Hu et al., 2020). In the brain, synaptic glutamate is taken up by astrocytes and converted into glutamine. Further, this glutamine is transported to neurons where it is converted into glutamate (Hertz et al., 1999; Magistretti and Pellerin, 1999; Deitmer et al., 2003). The known glutamine transporters belong to the SLC1, SLC6, SLC7 and SLC38 families. This system of transporters and enzymes involved in glutamine conversion and synthesis is often referred to as the glutamate/glutamine/GABA (GGG) cycle.

Glutamate imbalance in neurotransmission is a part of the pathophysiology and a hallmark of neurodegenerative diseases such as dementia, motor disorders, and neuro-developmental disorders (Chen et al., 2010; Maynard and Manzini, 2017). A number of genetic studies reported connections between mutations in SLCs and neurodegenerative and neurodevelopmental disorders such as autism spectrum disorder (ASD) (Chiocchetti et al., 2014), schizophrenia, bipolar disorder and obsessive-compulsive disorder (Nava et al., 2015). Another study reported Alzheimer’s disease (AD) associated to a pathological finding of glutamatergic receptor signaling, glutamate imbalance, and amino acid transport (Chiocchetti et al., 2014).

The SLC38 family consists of A and N system transporters. This family transports amino acids upward in their electrochemical gradient using Na^+^ ion as a driving force. Members of the SLC38 family play a critical role in the maintenance of amino acid homeostasis of the cell, and some members are known to regulate the mTOR signaling pathway, such as SLC38A2 and SLC38A9, and are critical for human health (Hundal and Taylor, 2009; Pochini et al., 2014; César-Razquin et al., 2015; Rebsamen and Superti-Furga, 2016; Menchini and Chaudhry, 2019). SLC38A2 (SNAT2) is classified as a system A family member and is extensively regulated by nutritional availability, hormonal signaling, and cellular stress and acts as an upstream amino acid sensor of the mTOR pathway. SLC38A2 was found to be involved in several medical conditions such as cancer, diabetes mellitus, epilepsy, and neurodegenerative diseases (Hundal and Taylor, 2009; Bröer and Palacín, 2011; Menchini and Chaudhry, 2019). SLC38A9 is located in lysosomes and acts as an arginine sensor to regulate mTORC1 and CASTOR protein through the Rag GTPas pathway (Jewell et al., 2015; Wang et al., 2015; Rebsamen and Superti-Furga, 2016). SLC38A3 and SLC38A5 have been suggested to play a role in the GGG cycle (Kandasamy et al., 2018), where they are responsible for the export of glutamine from astrocytes. The uptake of glutamine from astrocytes into neuronal synapses is most likely also controlled by one or several SLC38 proteins, most likely by SLC38A6 (Bagchi et al., 2014; Gandasi et al., 2021). SLC38A1, A4, A7, and A8 are also glutamine transporters expressed in neurons, although no specific function has yet been assigned to these (Schiöth et al., 2013; Hu et al., 2020).

The protein kinase complex mammalian target of rapamycin (mTOR) is a cellular hub that is highly important in neurology (Lipton and Sahin, 2014). The mTOR signaling is a central modulator of various cellular functions in all cells, such as protein synthesis, energy metabolism, lipid metabolism, mitochondrial biogenesis, and autophagy (Wolfson and Sabatini, 2017) and is a critical pathway for proper response to various stressors. mTOR regulates crucial physiological processes such as neurogenesis, differentiation, neuronal development, axonal and dendrite growth, synaptic plasticity and long-term potentiation in the brain.

Dysfunction in the mTOR pathway can results in increased protein synthesis, leading to increased cell growth and proliferation, which affects amino acid homeostasis (Lipton and Sahin, 2014; Takei and Nawa, 2014; Switon et al., 2017). Recently, a concept of mTORopathies has arisen as a demotion of signaling disorder implicated in neurodegenerative and neuropsychiatric diseases (Switon et al., 2017; Liu and Sabatini, 2020;Lipton and Sahin, 2014). Here, we focused on a solute carrier 38 family member 10 (SLC38A10), which is a bidirectional transporter of glutamine, glutamate, alanine and aspartic acid (Hellsten et al., 2017) and has not been well studied regarding amino acid balance and signaling in the brain. Different genetic studies have also indicated SLC38A10 as a potential drug target for various neurodegenerative and neurodevelopment disorders such as ASD (Celestino-Soper et al., 2011; Guan et al., 2019) as a novel mechanism to regulate glutamatergic signaling. In addition, SLC38A10 expression was lowered in AD brain sections and was proposed as a possible novel biomarker for AD (Arisi et al., 2011; Guan et al., 2019; Hashimoto et al., 2019). SLC38A10 is expressed in the ER and Golgi compartment in neuronal stem cells, mouse embryonic cells, and kidney cells and affects nascent protein synthesis {Tripathi et al., 2019}. Our recent study of SLC38A10 knockout primary cortex cells (KO) has established a connection with poor cell viability and p53 under oxidative and glutamate excitotoxicity stress (Tripathi et al., 2021).

To understand the role of SLC38A10 in amino acid balance and signaling in the brain, we used the SLC38A10 knockout mouse (KO) primary cells to investigate their role in metabolic and cellular signaling under nutrient stress. Primary cortex cultures (PCCs) from SLC38A10 KO mice show changes in protein synthesis regulation and reduced cellular cholesterol concentrations due to abnormal activation of mTOR signaling. KO PCCs subjected to amino acid starvation failed to modulate mTOR signaling to reduce protein synthesis and transcriptomic data show upregulated *TSC2* and downregulated *SERBP1* genes. Therefore, our data present a new finding of SLC38A10 as a glutamate regulator that modulates mTORC1 signaling, followed by affected protein and lipid synthesis in neurons and glutamate receptor signaling pathway. This possible underlying mechanism suggests a principal role of SLC38A10 in brain glutamate metabolism and neurodegenerative diseases.

## Results

### SLC38A10 is Involved in the Regulation of Intracellular Amino Acid Homeostasis

**FIGURE 1:**
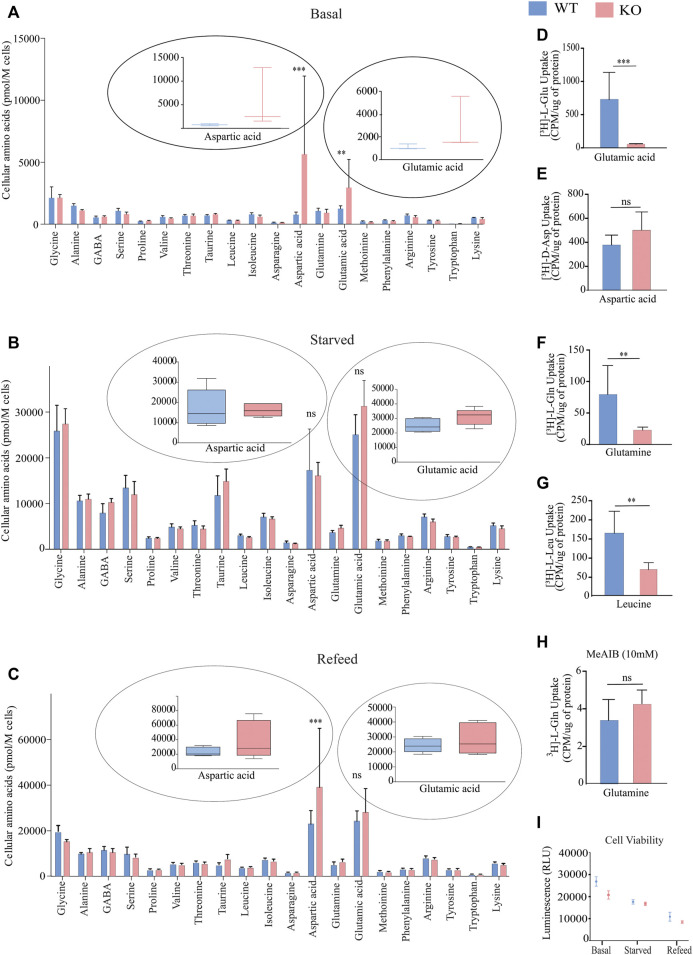
Measurement of intracellular amino acids and uptake in primary cortex cells deficient of SLC38A10 compared to WT using LC-MS/MS at basal, starved, and Refed conditions. **(A)** Intracellular amino acid levels in primary cortical neuronal cells (WT and KO) cultured in complete media for ten days. **(B)** The amino acid content in WT and KO PCCs starved in amino acids for 2 h after B27 deprivation overnight. **(C)** WT and KO PCCs refed with medium containing amino acids for 1 h after 2-h starvation of amino acids. **(A–C)** After culturing in different conditions, cells were washed, and the absolute levels of intracellular amino acids were quantified using LC/MS/MS. Data represent mean ± SD and *n* = 6. **(D–H)** Comparison of basal amino acid uptake in WT and KO primary cortical culture of 3H labeled amino acids under basal condition. Transport data of radiolabeled uptake assays were analyzed using 0.2 µM-radiolabeled substrates measured in counts per minute (CPM) using scintillation counter and normalized to total protein. The substrates investigated were: **(D)** glutamic acid, *n* = 8, *p* < 0.0001; **(E)** aspartic acid, *n* = 8, *p* = 0.0663; **(F)** glutamine, *n* = 8, *p* < 0.0001; **(G)** leucine, *n* = 8, *p* = 0.0071; and **(H)** glutamine in the presence of 10 mM MeAIB, *n* = 8, *p* = 0.0873. Data were analyzed using an unpaired *t*-test with 95% confidence interval (*p < 0.05, **p < 0.01, ***p < 0.001). The data illustrate the mean ± SD. **(I)** ATP concentrations were measured with the CellTiter-Glo Luminescent Assay during different treatment conditions: control, starved, and refed. For statistical analysis, an un-paired student t-test was performed.

SLC38A10 is an ER/Golgi localized transporter of glutamine, glutamate, alanine, serine and aspartic acid (Hellsten et al., 2017; Tripathi et al., 2019). To understand how SLC38A10 influences amino acid homeostasis in the brain, we measured intracellular amino acid concentrations in PCCs of WT and KO mice using liquid chromatography/tandem mass spectrometry (LC/MS/MS) under three different conditions: basal, amino acid starved and refed. At basal conditions, we measured 20 amino acids, with a significant increase in the concentration of aspartic acid and glutamic acid (Figure 1A). Interestingly, these differences disappear after starvation (Figure 1B). Furthermore, after refeeding amino acids to the starved cells for one hour, the aspartic acid and glutamic acid levels were reinstated (Figure 1C) as higher in KO PCCs.

### SLC38A10 Influences Uptake of l-Glutamine, l-Glutamate and l-Leucine

A radiolabeled uptake assay was performed to establish the uptake capacity for aspartic acid, glutamine, glutamic acid, leucine and system A selective inhibitor MeAIB in PCCs from KO and WT mice. The steady-state concentrations of radiolabeled amino acids were measured after a 60 min uptake of 0.2 M of the respective amino acid at a fixed concentration. Radiolabeled uptake of [^3^H]-L-Gln, [^3^H]-L-Glu, [^3^H]-D-Asp, and [^3^H]-L Leu in KRH buffer at 37°C. The KO PCCs displayed a lower uptake of L-GLn, l-Glu acid, and L-Leu (Figures 1D,F,G). However, no difference was observed in the uptake of d-Aspartic acid (Figure 1E). Further, a competition assay between 0.2 M of [^3^H]-L-Gln and unlabeled MeAIB was performed to estimate the ability of MeAIB to block [^3^H]-L-Gln uptake; however, the uptake of [^3^H]-L-Gln was not significantly different between the WT and KO cells. Since methylaminoisobutyric acid (MeAIB) acts as a blocker for system A transport (Tani et al., 2010), it suggests that system A transport is not affected in KO PCCs. To determine the effect of SLC38A10 knockout on energy regulation, we measured ATP at basal, starved, and refed conditions in PCCs from WT and KO mice. However, no difference was observed between the two genotypes (Figure 1I).

### Regulation of Amino Acid-Based mTORC1 Signaling in Response to Nutrient Starvation

**FIGURE 2:**
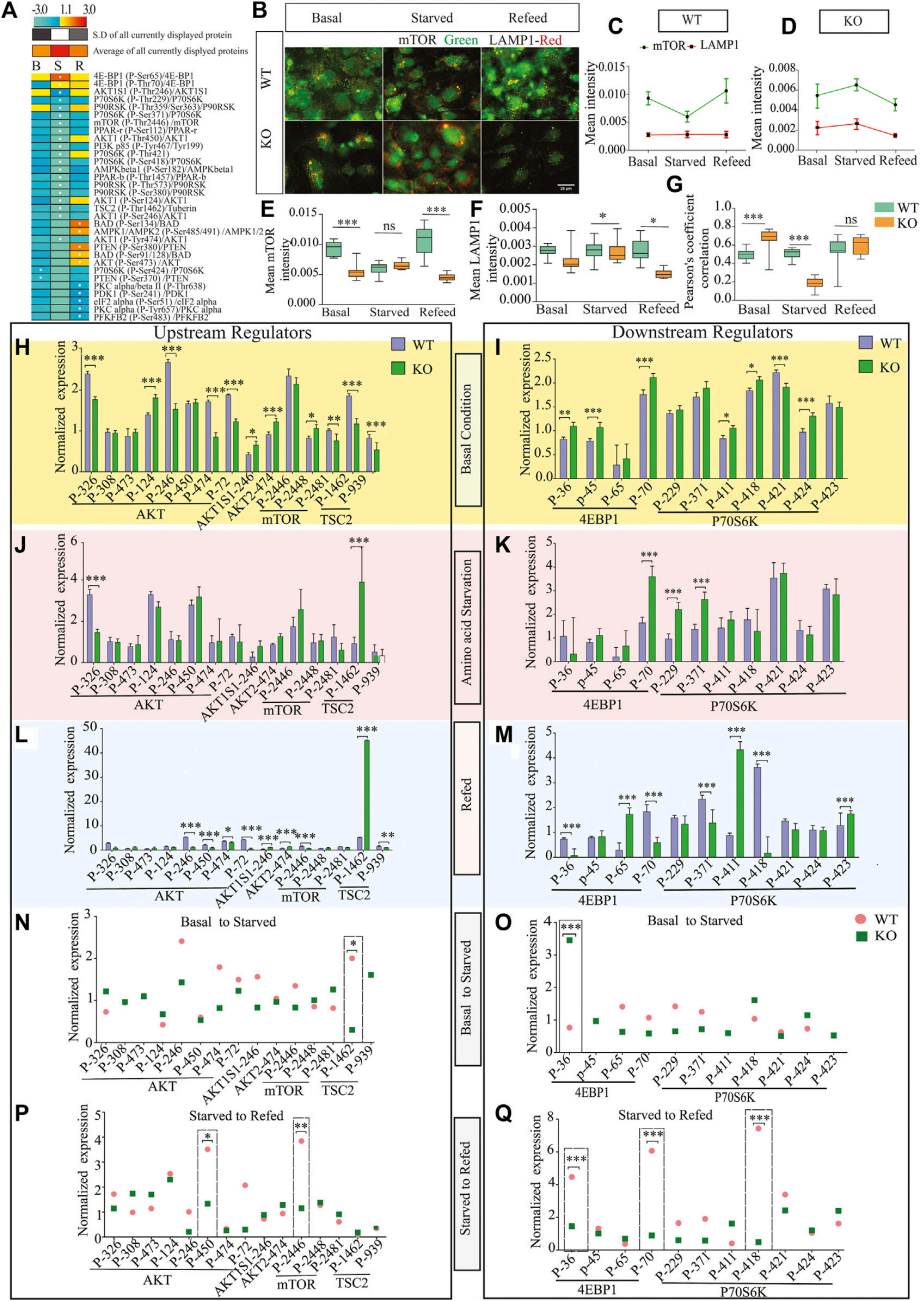
Dysregulation of the mTOR signaling pathway. **(A)**. Heat map representation of phosphoprotein ratio detected in full moon Biosystems mTOR phospho-protein array. Fold change was calculated after normalizing the average signal cor-responding to the median signal for each group, and the ratio has been calculated as KO *vs.* WT signals. Proteins that have shown at least 2-fold up or downregulation were included. **(B)**. KO cells show less co-localization of mTOR (green) with the lysosome (LAMP1, red) in refed and starved conditions than WT cortical cells. Scale bar represents 10 um. **(C,D)** The total intensity of mTOR and LAMP1 was measured with cell profiler using immunostaining images in WT **(C)** and **(D)** KO. Graphs represent mean ± SEM; *p < 0.05, **p < 0.01, Student’s t-test (*n* = 15–17 images for each condition). **(E–G)** Data representation of the total mean of intensity of **(E)** mTOR and **(F)** LAMP1 in an area of interest in the cytoplasm (doughnut) of WT cells at different conditions, respectively. Whispers max and min plot represent total mTOR and total LAMP1 intensity in the cell. **(G)** mTOR and LAMP1 correlation in basal, starved, and refed conditions. The graph rep-resents Pearson’s co-efficient correlation between LAMP1 and total mTOR in WT and KO cells. **(H–L)** Bar graph represents changed upstream protein phosphorylation changes at basal **(H)**, starved **(J)**, and refed **(L)** conditions. Further repre-sentation of phosphorylated target of downstream regulators altered at basal **(I)**, starved **(K)**, and refed conditions **(M)**. **(N,O)** Further targeted protein changed from the transition from basal to starved upstream regulators **(N)** and downstream regulators **(O)**. **(I,K)** represents affected proteins between trans ion from starved to refed, upstream regulators **(P)** and downstream regulators **(Q)**.

SLC38A10 is known to affect the rate of nascent protein synthesis (Tripathi et al., 2019), mainly controlled by the mTORC1 pathway. To understand the effect of the SLC38A10 transporter on the mTOR signaling cascade, which is primarily regulated by amino acid stimulus, we used the Full moon phospho-mTOR protein array (Ling et al., 2017). The mTOR array has a total of 138 sites and phospho-specific antibodies for molecules that participate in mTOR signaling. A comprehensive list of fold change expression in KO compared to WT PCCs under basal, starved, and refed conditions (Supplementary Table S1) was calculated. We also calculated how upstream regulators (AKT, mTOR, TSC2) and downstream regulators (4BP1, P70S6K) at basal (Figures 2H,I), starved (Figures 2J,K) and refed (Figures 2L,M) conditions in KO PCCs. Moreover, we calculated how these proteins respond to the transition between basal and starved states (Figures 2N,O) and the transition between starved and refed states. By doing this for KO and WT PCCs separately, differences in response can be analyzed (Figures 2P,Q). The phospho-protein ratio of each protein was calculated and presented in a heat map (Figure 2A).

Among these changed proteins, three proteins were differentially expressed between KO and WT in every condition: 4E-BP1 (P-Ser65), AKT1S1 (P-Thr246), P90RSK (P- Thr359/Ser363). To confirm some critical findings from the mTOR antibody array, we performed western blots to measure the total mTOR (Figures 2H,I), phosphorylated mTOR (p-thr2448) (Figures 2H,J,K), downstream protein S6 (Figures 2H,M), phosphorylated p- S6 (Ser240/244) (Figures 2H,N,O), TSC2 (Figures 2H,L), and p70S6 (Figures 2H,P) for basal, amino acid starved, and refed conditions. KO PCCs show upregulation in total mTOR, p-mTOR (2448), and total S6 ribosomal protein during amino acid starvation compared with WT PCCs, whereas at basal and refed conditions, p-S6 (Ser240/244) was more expressed in KO PCCs. The upstream regulator TSC2 is higher in KO cells than in WT cells in basal conditions but not in starved and refed conditions. There was no significant difference in p70S6K protein between KO and WT cells, which does not correspond with the array data.

By analyzing the transitions between the three conditions (Figure 2N) for proteins involved in an upstream regulator, we showed that from basal to starved, the TSC2 phosphorylation of residue 1462 was significantly differently affected in KO PCCS compared to WT in a one-way ANOVA analysis (*p* > 0.05). Similarly, in the transition from starved to refed, the phosphorylation of upstream regulator AKT (residue 450) and mTOR (site 2446) had significantly different responses in KO PCCs compared to WT. Also, the downstream regulators 4EBP1 (sites 36 and 70) and p70S6K (site 418) differed between KO and WT PCCs. Western blot was performed on some of the proteins represented in Supplementary Figure S2.

**FIGURE 3:**
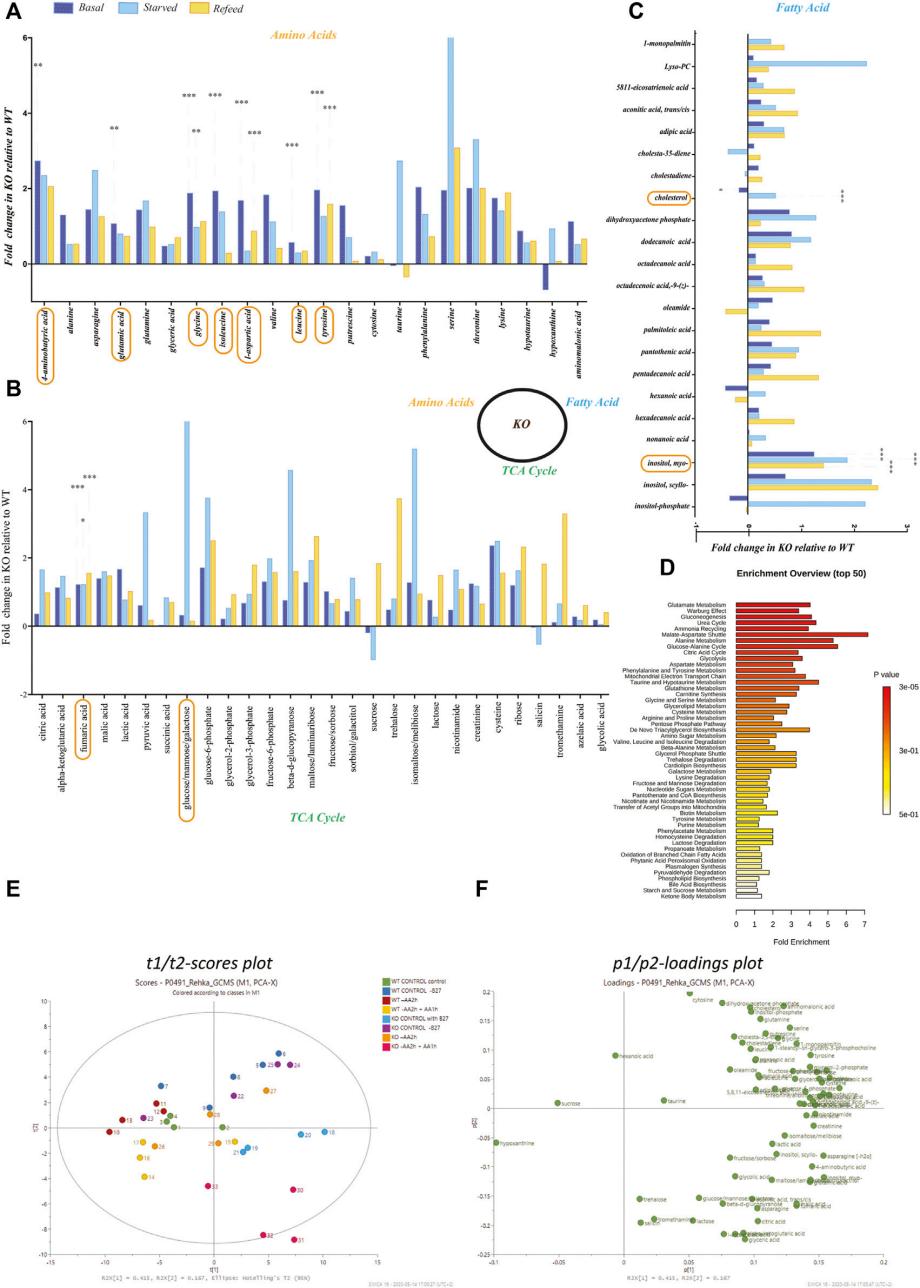
Metabolomics profiling of amino acids, fatty acids, and TCA/glycolysis intermediates in KO primary cortex cells. Metabolomics was performed on WT and KO PCCs using GCMS-MS. **(A–C)** Fold change of amino acids in KO compared to WT primary cortical neurons for **(A)** amino acids, **(B)** TCA cycle metabolites, and **(C)** metabolites involved in the fatty acid synthesis. Statistical analyses were performed, and significantly changed molecules were marked after performing a two-way ANOVA and Bonferroni post-tests (*p < 0.05, **p < 0.01, ***p < 0.001). **(D)** Metaboanylate pathway enrichment of detected metabolites between KO and WT PCCs. Here, we only show metabolite pathways with at least three metabolites in the set. **(E,F)** PCA plots based on the metabolomics profile of KO and WT PCCs. **(E)** Two dimensional t1/t2-scores plot and **(F)** loadings plot.

Pathway analysis was performed using Ingenuity Pathways Analysis (IPA) software and using the Molecule Activity Predictor (MAP) function; prediction indicated increased protein translation in KO PCCs (Supplementary Figure S1- A (basal), F (starved), E (refed)). Comparison analysis between treatments uncovers the list of upstream and downstream molecules of PI3K/AKT/TOR pathway and p70S6 signaling pathway was significantly affected in KO PPCs (Figures 3B,C) at basal, starved and refed states. Regulation of mTOR and p70S6K signaling affected KO cells, which remain upregulated in starved conditions compared to WT PCCs. Results from disease and molecular function indicated an increased sensitivity to neurological diseases in KO due to an absence of SLC38A10. Dysfunctional mTOR signaling increases the prediction of neurological disorders associated with AD, abnormal morphology of the nervous system and schizophrenia (Supplementary Figure S1F).

### Role of SLC38A10 in Amino Acid-dependent mTOR Signaling Under Nutrient Stress

Lysosomal mTOR localization gets affected by nutrient-dependent mTOR signaling. Additionally, to evaluate the relationship between the activity of LAMP1 and mTOR and its intracellular positioning in KO PCCs, we performed immunostaining of LAMP1 and mTOR under control, starved and refed conditions. It showed co-localization of the two mTOR and LAMP1 proteins (Figure 2B), indicating that in starved conditions, mTOR movement from the lysosome influences the amino acid starved disease in KO PCCs (Figures 2B–G) in KO PCCs. To study this, we plotted the total mean intensity of mTOR and LAMP1 in graphs separately for WT and KO cells (Figures 2C,D). The intensity of LAMP1 and mTOR is a relation test, which shows a significant (*p* < 0.0001) modulation of co-localization of LAMP1 and mTOR in KO PCCs compared with WT under control and starved condition (Figure 2G). Our findings reveal that at starved conditions, localization of LAMP and mTOR is affected and maintains active mTOR signaling in KO PCCs. It indicates the impaired activity of mTOR signaling and affected lysosomal mTOR localization due to the absence of SLC38A10 in PCCs.

### The Absence of SLC38A10 Affect the TCA and Fatty Acid Synthesis Cycle in PCCs

We performed untargeted metabolomics on WT and KO PCC, and 72 metabolites were detected (Figures 3A–C). Metabolomic data show a change in the following metabolites at a basal level between WT and KO PCCs: l -aspartic acid (*p* < 0.001), tyrosine (*p* < 0.001), fumaric acid (*p* < 0.001), glycine (*p* < 0.001), isoleucine (*p* < 0.001), Myo-inositol (*p* < 0.001), leucine (*p* < 0.001), glutamic acid (*p* < 0.01), GABA (*p* < 0.01), and cholesterol (*p* < 0.05) (Figures 3A–C). After complete amino acid starvation for two hours, five metabolites (fumaric acid (*p* < 0.05), glucose (*p* < 0.05), glycine (*p* < 0.01), Myo-inositol (*p* < 0.001), and cholesterol (*p* < 0.001)) were changed in KO PCCs compared to WT. After refeeding cells with amino acids, altered levels of six metabolites (fumaric acid (*p* < 0.001), glycine (*p* < 0.01), Myo-inositol (*p* < 0.001), tyrosine (*p* < 0.001), and l-aspartic acid (*p* < 0.001) (Figures 3A–C) were found. There were no significant changes observed in cholesterol in the refed group (Figure 3C).

### Transcriptional Change of Solute Carrier Transporters Leads to an Effect on Neuronal Function

**FIGURE 4:**
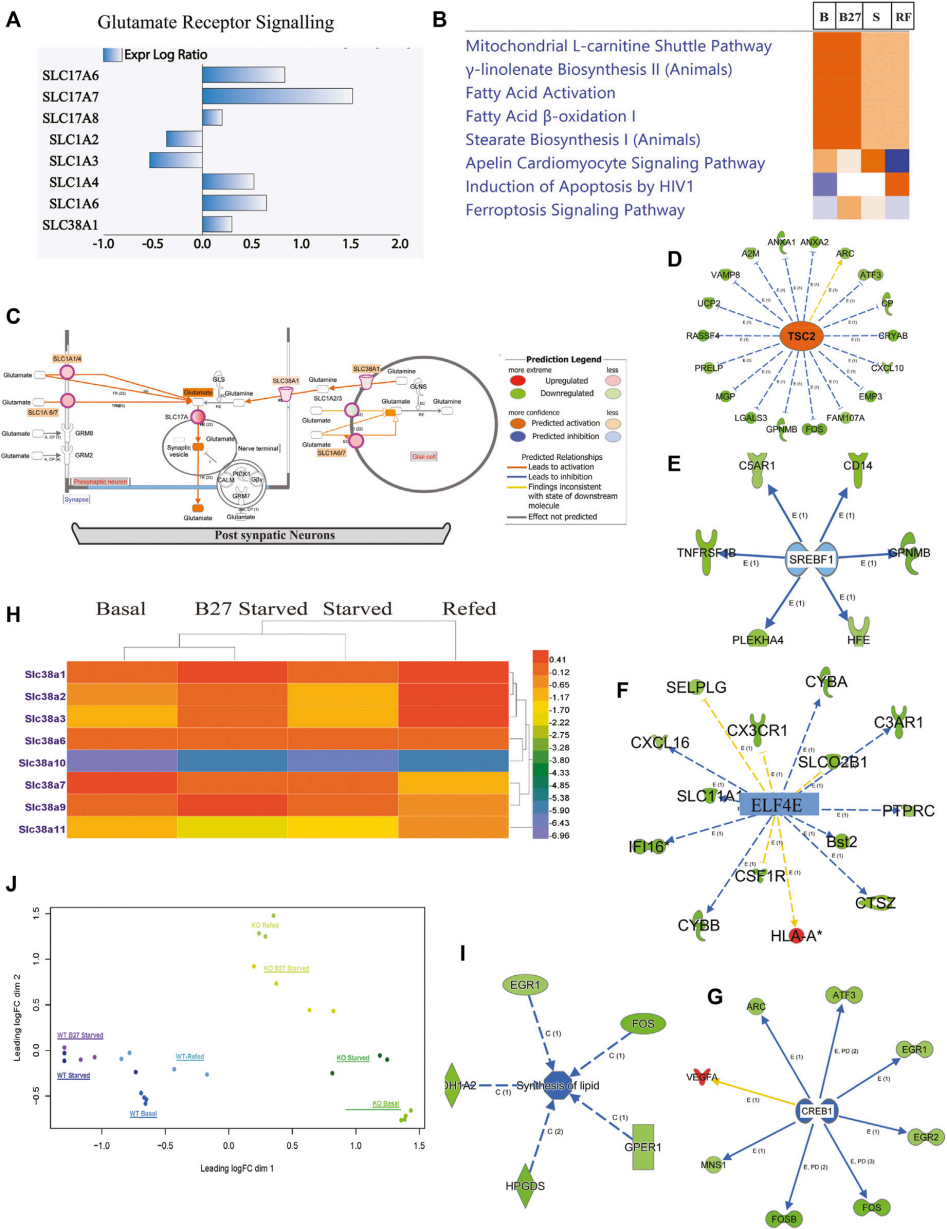
Expression of genes involved in amino acid transport, fatty acid synthesis, and glutamate receptor signaling in KO PCCs. At basal, starved, and refed conditions. Solute carrier transporters changed at basal **(B)**, starved (S), and refed (R) condi-tions separately, analyzed by IPA software. KO *vs*. WT fold change using pathway analysis IPA software. Cut-off values were selected as minimum two-fold downregulated or upregulated (−2< FC > 2, adjusted *p*-value < 0.05) in the KO com-pared to WT cells. **(A)** Solute carrier transporter transcripts changed, involved in glutamate receptor signaling. **(B)** Comparison analysis of changed expression of SLCs gene at basal **(B)**, overnight B27 starved (B27); amino acid starved for 2 h (S), and amino acid refeeding for 1 h (RF). **(C)** Representation of glutamate receptor signaling using Molecule Activity Predictor (MAP): activation of pathway predicted at basal in KO PCCs. **(D)** Representation of gene changes predicting accelerant TSC2 expression in KO PCCs at basal level. **(E)** Representation of gene changes predicting inhibited SREBF1 expression in KO PCCs at basal level. **(F)** Representation of gene changes predicting inhibited ELF4E expression in KO PCCs at basal level. **(G)** Number of gene changes predicting inhibited CREBP1 expression in KO PCCs at basal level. **(H)** Heatmap members of SLC38 family transporter altered in KO PCCs at different B, B27, S, and RF conditions. Data represented fold change in KO PCCs compared to WT. **(I)** Gene alterations affected lipid synthesis at basal levels. **(J)** Principal component analysis (PCA) plot of WT and KO samples at different treatment level conditions: B-Basal, B27-B27 starved, S-Amino acid Starved, and RF-Refed.

**FIGURE 5:**
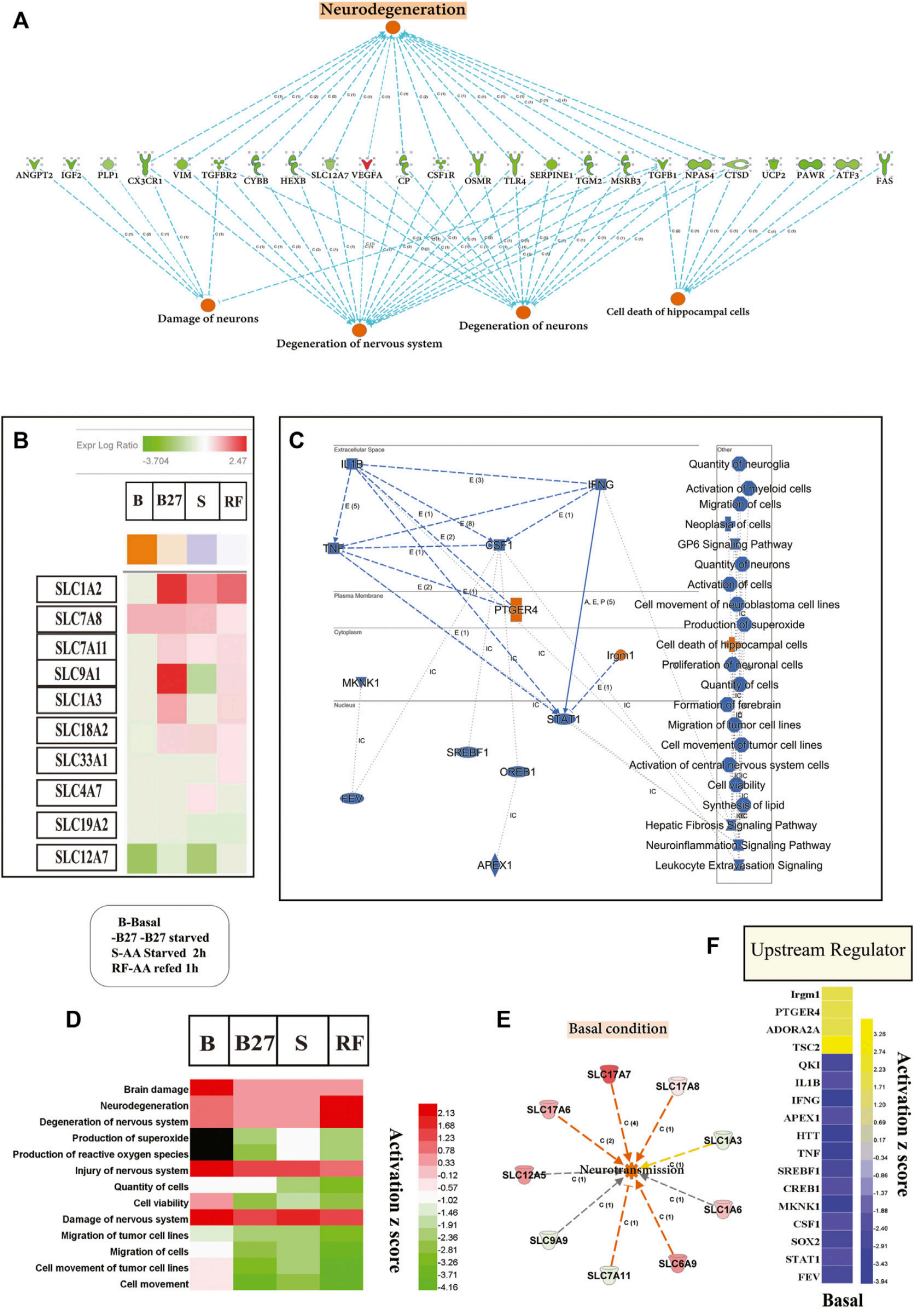
The absence of SLC3A10 leads to transcriptome changes of genes in KO PCCs involved in modifying cellular, molecular functions and indicating neurological disease. Complete transcriptomic data and changed gene list subjected to IPA software and focused analysis performed on neurological function and disease. **(A)** Representation of genes from a basal level of transcriptomic data indicated a rise in neurodegeneration of KO PCCs **(B)**. Heat-map representation of solute carrier transporter involved in neurodegeneration and affected expression under B, B27, S, and RF levels. **(C)** Repre-sentation of overall summary changes of molecules of Transcription regulator (TR), cytokine-Kinase-K- Enzyme- E, G-protein coupled receptor-GPCR, at a subcellular level, cellular, molecular functions, and cellular pathways in the basal level of KO PCCs. **(D)** Heat-map represents comparison analysis using IPA focused on disease, and cellular function at B, B27, S, and RF conditions accordingly activated Z scores. **(E)** At altered basal expression, SLC enhances neurotransmis-sion function in KO PCCs. **(F)** The heat map of top upstream molecules accordingly activated Z-scores, resulting in in-hibition and upregulation prediction at the basal level. Transcription regulator (TR), cytokine-Kinase-K- Enzyme- E, G-protein coupled receptor-GPCR. Upstream regulator analysis was performed using IPA.

**FIGURE 6:**
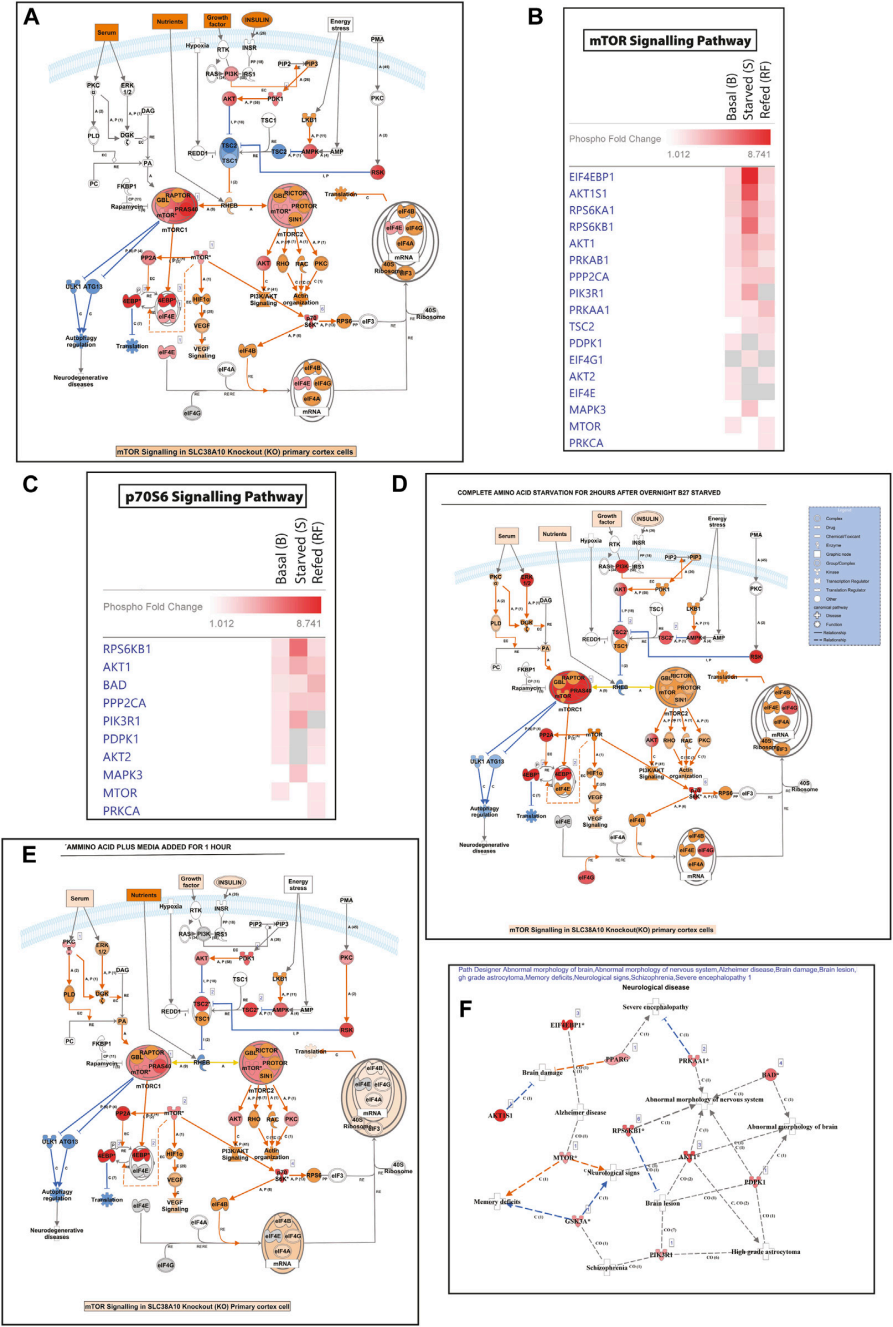
The response in mTOR pathway and amino acid regualtion following SLC38A10 knockout. Complete transcriptomic data and changed gene list subjected to IPA software analysis. **(A)** Regulation in the mTOR pathway in response to SLC38A10 knockout in Basal condition, blue symbols indicate down regulation and red symbols up regulation. **(B)** Heatmap describing changes in transcript levels from the mTOR pathway during Basal, Starved and Refed conditions. **(C)** Heatmap describing changes in transcript levels from the p70S6 pathway during Basal, Starved and Refed conditions. **(D)** Regulation in the mTOR pathway in response to SLC38A10 knockout in Starved condition, blue symbols indicate down regulation and red symbols up regulation. **(E)** Regulation in the mTOR pathway in response to SLC38A10 knockout in Refed condition, blue symbols indicate down regulation and red symbols up regulation. **(F)** Neurological diseases predicted by Path Designer, in response to SLC38A10 knockout.

We performed an ampliseq transcriptomic analysis of PCCs from KO and WT mice under four conditions: basal (B), overnight B27 starvation (B27), amino acid starvation (S), and refed condition (RF) (SE1, Figures 1–6). Principal component analysis (PCA) was performed to study how KO cells were globally different from WT in response to treatments, B, B27, S, RF (Figure 4J), which shows clustering of genotype and condition, but minimal clustering of the same treatment of the two genotypes. After normalization (Supplementary Datasheet S1), highly regulated differentially expressed (DE) gene lists, along with affected SLCs (Supplementary Datasheet S1), were extracted and used in enrichment pathway analysis, based on known molecular interactions in IPA, which defines molecular patterns that provide insight into the possible molecular interactions.

We performed a focused analysis on the primary cells and nervous system and included only SLCs with cut-off ≥2-fold change and FDR-adjusted *p* ≤ 0.05 under B, B27, S, and RF conditions. SLCs affected in all four states were used for canonical pathway analysis. In KO PCCs, 269 SLCs were two-fold upregulated at the transcriptional level (SE1) at basal conditions. Changes in SLC exprssion predict an effect on cellular functions, e.g. mitochondrial pathway, fatty acid pathway, transport of molecules and neurotransmission (Figure 5B). Most affected canonical pathways changed in KO PCCs under these four conditions are shown in Figure 4B, expression of genes involved in glutamate receptor signaling (Figures 4A,C), mitochondrial l-carnitine shuttle pathway and fatty acid synthesis (Figure 4B). In addition, IPA software molecular predictor activity (MAP) function indicates upregulation of SLCs (Figure 4A), which shows an increase in the concentration of glutamate in synapse as a result of upregulation of SLC1A2/3, SLC1A6/7 in glial cells and SLC17A, SLC1A1/4, SLC1A6/7 (Figure 4C, basal level).

Members of the SLC38 family changed expression differently under B, B27, S, and RF conditions, i.e., SLC38A1, SLC38A2, SLC38A3, SLC38A6, SLC38A7, SLC38A9, and SLC38A11 (Figure 4H and Supplementary Datasheet S2). Interestingly, at basal level, SLC38A3 and SLC38A11 showed 50% downregulation in KO PCCs. At starvation, SLC38A2, SLC38A3, and SLC38A11 were downregulated 50%. Other affected members of the SLC38 family were SLC38A1, SLC38A6, SLC38A7, and SLC38A9, which were altered at control, starved and refed conditions (Figure 4H). An upregulation of SLCs is indicative of having a substantial effect on the list of cellular activities at all conditions (Figure 4B), transport of molecules, as well as on neurotransmission of neuronal cells (Figure 5E), fatty acid synthesis (Figure 4I), and glutamate receptor signaling (Figure 4C). Upstream analysis shows inhibition of EIF4G2 (Figure 4F), which is the regulator of 5 cap-dependent protein translations.

Further investigation of the effect of alters genes effect on KO PCCs at basal condition using the list of complete gene list (Supplementary Datasheet S1) showed inhibition of regulator sterol regulatory element-binding transcription factor 1 (SREBF1) and decreased lipid synthesis in KO PCCs (Figures 4E,I). Another negative regulator of mTOR, TSC2, was upregulated, affecting downstream mTOR signaling in PCCs (Figure 4D). Another regulator, cAMP Responsive Element Binding (CREB1) protein, was inhibited, an indicator of ER stress and modulates the unfolded protein response. After upstream analysis, top activation regulators IRGM1, PTGER4, ADORA2A and TSC2 were higher than the z-score in basal condition. In contrast, interferon Gamma (IFNG), tumor necrosis factor (TNF), Huntington’s disease protein (HTT), MAPK Interacting Serine/Threonine Kinase 1 (MKNK1), transcription Factor SOX2 (SOX2), FEV, QKI, SREBF1, Interleukin 1 Beta (IL1B), CSF1, APEX1, STAT1, CREB1 were inhibited (Figure 5F and Supplementary Table S1).

### Loss of SLC38A10 Results in Changes in Brain Metabolism Predicted to Induce Neurological Disease

Transcriptomic changes show that molecular, cellular and biological processes affect neuronal degeneration and neurological disease (Figures 5A,D) and indicted top upstream regulators involved in these changes (Figure 5C). At basal condition, KO PCCs show increased neuronal damage, degeneration of the nervous system, and increased cell death of hippocampus cells, which signal an increased risk of neurodegenerative disease. Several cellular functions altered in KO PCCs predict a decrease in neuronal cell viability, affecting the neuro inflammation-signaling pathway (Figure 5C). Various cellular processes altered between the genotypes under all conditions (B, B27, S, and RF) indicate upregulated cellular pathways in degeneration such as brain damage, damage to the neurons system, and increased neurodegeneration (Figure 5D). Pathway analysis using changed SLC genes shows a dysregulated expression of SLC1A2, SLC7A8, SLC7A11, SLC9A1, SLC1A3, SLC18A2, SLC33A1, SLC4A7, SLC19A2 and SLC12A7 represented as a heatmap of four conditions to predict an increased tendency of neurodegenerative disease in KO cells (Figure 5D; Supplementary Table S3). At basal state, changes in expression of SLCs suggested an increased rate of neurotransmission (Figure 5E) in KO PCCs. The detailed investigation at basal condition lists metabolic disease, organismal injury, and abnormalities. In KO PCCs, various molecules’ increasing sensitivity to AD were identified; specifically molecules predicted to increase beta amyloidosis and cerebral amyloid angioplasty (Supplementary Table S2).

## Discussion

SLCs provide crucial regulation of brain glutamate homeostasis. In this study, we uncover the unknown function of the ER/Golgi localized SLC38A10, an evolutionary old SLC that transports glutamate, glutamine and aspartic acid in the brain and regulates protein synthesis and plays a role in p53 mediated cell survival under stress (Hellsten et al., 2017; Tripathi et al., 2019; Tripathi et al., 2021). We demonstrate that SLC38A10 is essential for the uptake of glutamine and glutamate, and also important for PI3K/AKT/mTOR-dependent protein synthesis. To understand amino acid growth factor-dependent mTOR signaling, KO PCCs subjected to amino acid starvation resulted in affected phosphorylation of upstream and downstream regulators. Previous studies on TSC2 knockdown resulted in dysregulated AKT/mTOR signaling (Choi et al., 2008; Appenzeller-Herzog and Hall, 2012; Nie and Sahin, 2012; Lipton and Sahin, 2014). We found an increased degree of phosphorylation of 4BP1 and P70S6K1, indicating upregulation of protein synthesis. In neurons, 4BP1 plays a role in cap-dependent mRNA translation in axons and protein synthesis in synapses (Khoutorsky et al., 2015; Kapur et al., 2017; Roux and Topisirovic, 2018). Our study shows that SLC38A10 loss affected AKT/TSC2/mTOR regulation and resistance to nutrient stress. It is known that affected TSC2 regulation increases cell stress-resistant and hyperactive mTOR (Inoki et al., 2003; Smith et al., 2005; Di Nardo et al., 2009; Han and Sahin, 2011; Demetriades et al., 2014; Guenther et al., 2014; Rozas et al., 2015).

With a series of biochemical, transcriptomic and metabolomic experiments performed on KO PCCs, we show that the SLC38A10 acts as a critical glutamate homeostasis modulator in brain cells. We observed increased intracellular glutamate, aspartic acid and reduced glutamate and glutamine uptake; we also measured increased intracellular glucose concentration at basal conditions. Glutamate is the major excitatory neurotransmitter, maintaining the glutamate-glutamine cycle. SLCs and various receptors tightly control synaptic glutamate concentration to protect cells from excitotoxicity and prevent synaptic dysfunction (Zhou and Danbolt, 2014).

Our transcriptomic study corroborates alterations in the glutamate regulating systems in SLC38A10 KO PCCs illustrating expression changes of other glutamate SLCs such as SLC1, SLC7, and SLC38 family (Figure 4H), which have a role in maintaining the glutamate pool in the neuronal-astrocyte network (Kanai and Hediger, 2003; Tani et al., 2014). Polymorphisms in SLCs regulating glutamate have been demonstrated to be involved in many neurodegenerative and neurodevelopmental diseases, e.g., SLC1 family members (Sheldon and Robinson, 2007; Basu et al., 2009; Damseh et al., 2015). Consequently, changed expression of genes indicating increased neurotransmission in KO cells suggests that the SLC38A10 transporter could be involved in regulating the GGG cycle in the brain (Figures 5C, 6E), which could result in disturbed neuronal homeostasis. In KO PCCs, pathway analysis highlights strong effects on upstream transcription regulators, in particular TSC2. In our data, TSC2 is inhibited, and earlier studies with TSC2-deficient neurons show mTORC1 hyperactivation despite increased autolysosome accumulation and retained autophagy flux (Lipton and Sahin, 2014).TSC2 is an upstream regulator of the master regulator mTOR, controlling lipid and protein synthesis (Switon et al., 2017). At basal culturing conditions, KO PCCs show increased expression of protein translation regulator ELF4E.

In KO PCCs, metabolomic and transcriptomic data indicated decreased fatty acid synthesis by inhibiting SREBP2, predicted to result in lower cholesterol levels at basal conditions (Laplante and Sabatini, 2013). SREBP-2 is located in the ER, and mTORC1 triggers translocation from ER to the Golgi to produce mature SREBP-2 and move to the nuclear transcription factor. SREBP-2 activation is associated with lower ER cholesterol levels and is inversely restrained by mTORC1 activity (Eid et al., 2017). Earlier histopathological high throughput screening of SLC38A10 knockout mice relieves its associated function with transportation of lipid, amino acid (protein) and minimum hepatic lipidosis, which is shown as overall growth retardation (Adissu et al., 2014).

Further, we explored the possible role of SLC38A10 as an amino acid-sensing member of the SLC38 family, similar to the transceptors SLC38A2 and SLC38A9. We performed an amino acid starvation experiment to understand LAMP localized mTOR signaling in KO PCCs. We hypothesized that SLC38A10 acts as a transceptor for mTOR signaling in response to glutamate concentration in cortex cells. We propose a model where SLC38A10 acts as a glutamate transceptor to sense glutamate and modulate mTOR signaling. Our data is consistent with the role of SLC38A10 as sensors of glutamate homeostasis in the cell using the knockout model, which gets imbalanced because of the absence of this transporter. It fits well with the model for metabolite sensing and signaling with the role of transporters as transporter and receptor (Hundal and Taylor, 2009; Kriel et al., 2011; Eid et al., 2017; Wang and Lei, 2018). SLC38A10 is localized to ER and Golgi and has a predicted structure with an evolutionary conserved unusually long extracellular C-terminal of 722 amino acid residues (Hellsten et al., 2017). This conserved region of SLC38A10 could be involved in cell signaling functions, as many transceptors studied affect subcellular expression, stability and cellular signaling (Jewell et al., 2015; Mikros and Diallinas, 2019).

Mammalian target of rapamycin (mTOR) signaling plays a critical role in regulating activity-dependent protein synthesis in neurons. Data from the mTOR protein array and western blot show dysregulated mTOR signaling at basal, starved and refed conditions in KO PCCs. At the basal level in KO PCCs, phosphorylation of the repressor of mRNA translation and eukaryotic initiation factor 4E-binding protein 1 (4E-BP1) is increased. This should further activate S6 kinase (S6K1 or p70S6K), which in turn phosphorylates the ribosomal protein S6 (Maiese et al., 2013; Shimobayashi and Hall, 2015). Protein kinase P90RSK is known to regulate synaptic protein synthesis (Angenstein et al., 1998). TSC2 inhibition is known to inhibit mTOR and protein synthesis, which causes abnormal axonal regeneration in neuronal cells (Choi et al., 2008; Han and Sahin, 2011; Ru et al., 2012). Data from the full moon phosphorylation array, together with western blot data, suggest that starvation and refeeding are strongly blunted, with an inadequate response to these changed conditions in KO PCCs regarding mTOR phosphorylation. This is true for several upstream and downstream molecules at several phosphorylation sites, suggesting that among other functions, protein synthesis is not reduced in response to starvation and that this system is not reinstated in response to refeeding. This supports the conclusion that SLC38A10 is a sensor for, nutritional conditions and nutritional stress and that the cells cannot handle these situations properly in the absence of SLC38A10.

We also study co-localization of LAMP and mTOR at basal, starved and refed conditions to assess if it affect amino acid starvation and regulates mTOR signaling. Recent data show that multiple proteins which reside in the cytosol and on the lysosomes engage in the recruitment of mTORC1 to the lysosome (activation) and its release from the lysosome (inactivation) (Averous et al., 2014; Demetriades et al., 2014; Puertollano, 2014; Demetriades et al., 2016). It is also known that different amino acids have different roles in recruiting mTOR to lysosomes (Averous et al., 2014). As we found increased glutamate and changed mTOR signaling, we explored the effect of SLC38A10 removal on the lysosomal localization of mTOR. We reasoned that in KO PCCs, vacuolar H^+^ ATPase-dependent lysosomal dysregulation results in an acidification defect in certain neurodegenerative diseases (38), which would affect the proper movement of the mTOR complex. Furthermore, KO cells have increased aspartic and glutamic acid concentrations, which indirectly affect lysosomal-based mTOR localization. We conclude that the absence of SLC38A10 has resulted in the removal of glutamate/aspartate sensor in the cell, which affects lysosomal coordinated mTOR localization in cells causing dysregulation (Beugnet et al., 2003; Taylor, 2014; Chantranupong et al., 2015; Wang et al., 2015; Diallinas, 2017; González and Hall, 2017).

Our recent article showed increased cell death in SLC38A10 KO PCCs at the basal level. In contrast, oxidative and glutamate toxicity resistance suggests that KO PCCs are exposed to high glutamate levels under normal conditions, upregulating mechanisms to protect from glutamate-induced excitotoxicity. We have also found mitochondrial dysfunctionality and lower p53 expression in KO PCCs (78). Taken together, this observation and our finding confirmed a report showing dysregulation of glutamate homeostasis and mTOR signaling in KO PCCs resulting in neurodegeneration (Diallinas, 2017). Abnormalities of SLCs involved in regulating synaptic signaling and glutamate homeostasis give rise to excess glutamate levels, possibly resulting in excitotoxicity, which is a significant factor in the initiation and progression of neurodegenerative disorders, including ALS, AD, and Huntington’s disease (Lewerenz and Maher, 2015). Earlier work on AD brain lesions detected lower expression of SLC38A10 and predicted it to be a potential plasma biomarker of AD (Hashimoto et al., 2019). Furthermore, genetic mutation of SLC38A10 has been found to be associated with schizophrenia and bipolar disorder (Guan et al., 2019).

Here, we establish a transceptor model of SLC38A10, glutamate sensing, and amino acid-dependent mTOR signaling to regulate protein synthesis and fatty acid synthesis in brain cells (Hundal and Taylor, 2009; Chantranupong et al., 2015; Wang and Lei, 2018). SLC38A10 is crucial for a healthy brain function; it maintains glutamate homeostasis and provides a proper response to nutrient sensing.

In conclusion, the SLC38A10 knockout model revealed the new finding of SLC38A10 in glutamate metabolism, mTOR dependent protein and fatty acid regulation, supporting a previous association between SLC38A10 polymorphisms and neurodegenerative diseases.

## Materials and Methods

### Animals

The animal experiments were approved by the Uppsala Animal Ethical Committee (3 5.8.18–09820/2018) and followed Swedish legislation on animal welfare. The animals were kept in an animal facility with a controlled environment, including humidity (45–65%), temperature (20–24°C), and ventilation system and with a 12-h light/dark cycle with lights on at 7 a.m. In this study, a complete constitutive knockout was used. The technology used to create the KO mouse was the knockout-first allele approach (further described by Testa et al., 2004), and the KO mice (B6Dnk; B6N-Slc38a10^tm2a(EUCOMM)Wtsi^/H) were bought from the International phenotyping Consortium (IMPC). The mice were continually bred on a C57BL/6 background.

### Primary Embryonic Cortex Culture

Homozygous male and female mice were put together in the afternoon. The presence of a vaginal plug was checked the morning after. The morning when a vaginal plug was detected was defined as gestation day 0.5. On embryonic day 15.5, female mice were killed through cervical dislocation, and embryos dissected out and decapitated. Cortices were dissected and used to generate primary cortex cultures (PCCs), according to (Tripathi et al., 2021; Hellsten et al., 2018; Tripathi et al., 2019).

Briefly, e15 embryos were removed and kept in cold HBSS (Gibco). Dissected cortices were pooled after dissection and washed in 1X PBS with 10 mM glucose. The tissue dissociated following both chemical and mechanical procedures an enzymatic mixture of DNAse (Invitrogen) (1ul) and papain (Sigma) (10ul) in 1 ml diluted in PBS-10mM glucose for 20–30 min at 37°C, 5% CO2. Enzymatic digested cell suspension in plating media containing DMEM/F12 (Invitrogen), 10% FBS (Gibco), 2 mM GlutaMax (Invitrogen), 1 mM Na-pyruvate (Invitrogen) and 1% Penicillin/streptomycin (Invitrogen). The cell suspension was filtered using a 70 μm cell strainer (BD Stockholm, Sweden) and counted with a hemocytometer to ensure single-cell suspension for cell seeding.

Cells were incubated for 3 h at 37°C, 5% CO2. Once the cells were attached, Neurobasal media containing Neurobasal A media (Invitrogen), 2% B27, 1% Penicillin/streptomycin, and 2 mM GlutaMax and 1 mM Na-pyruvate replaced the media. Two days after plating, half of the media was replaced with fresh media, cells cultured for total ten days. The plates/coverslips (12mm, Menzel Glaser) to be used were coated with poly-l-lysine (Sigma-Aldrich). Cells were plated at a density of 10 × 10^3^ cells/well for 96 well plate; 50 × 10^3^ cells/well for 24 well plate; 7 × 10^5^-10^6^cells/well for six-well (Nunclone delta, Thermo Fisher Scientific) plate.

### Amino Acid Starvation and Refeeding Protocols in Primary Cortex Cells

Primary cortex neuronal cells were grown in 6-well plates for ten days. Starvation media replaced complete media, and cells were starved for 2 h at 37°C. The starved media was prepared using DMEM without amino acids (US Biologicals, D9811-16) and 1% Pen-strep (Gibco, Invitrogen). Refeeding experiments were carried out using refeeding media (amino acids plus media; +AA) for 60 min after 2 h of complete amino acid starvation (-AA). Refeeding media prepared using 100 × stocks of MEM amino acid mixtures and 50X MEM NEAA were added to 1X concentration of DMEM without amino acids (Starvation media) to the final concentration as found in DMEM media. For metabolites and amino acid detection, the cell pellet was collected and washed three times, with PBS, and excess PBS removed, and the cell was stored at 80°C and sent to the Umea Metabolomics centre. Cells were grown on a coverslip in 24 well plates, and starvation and refeeding experiment performed as described above and fixed using 4% PFA and further used for immunocytochemistry. For western blot, 1 × 10^6^ cells were seeded per well in 6 well plates. Cells were further subjected to starvation and refeeding experiment and cell lysates were prepared for the immunoblot.

### LC/MS/MS Measurement of Intracellular Free Amino Acids

Amino acid extraction -One million cells were extracted with 400 µL of the same extraction solution, one tungsten bead was added, and the samples were shaken at 30 Hz for 2 min in a mixer mill. After removing the bead, the samples were centrifuged at 4°C, 14000 rpm, for 10 min. From both samples, the supernatant was collected and stored at −80°C until analysis.

#### Amino Acids Derivatisation With AccQ•Tag

According to the manufacturer’s instructions, extracted samples were derivatised in AccQ•Tag™ (Waters, Milford, MA, United States). Briefly, ten10 μL of the extract was added to 70 μL of AccQ•Tag Ultra Borate buffer, and finally, 20 μL of the freshly prepared AccQ•Tag derivatisation solution was added, and the sample was immediately vortexed for 30 s. Samples were kept at room temperature for 30 min, followed by 10 min at 55°C. For each batch quality control sample and procedure, blanks were included. Calibration curves were prepared similarly by adding 10 µL of each point of the curve to 70 µL of AccQ•Tag Ultra Borate buffer and 20 μL of the freshly prepared AccQ•Tag s derivatisation solution.

### Amino Acids Quantification by LC-ESI-MSMS

Derivatised solutions were analysed using a 1290 Infinitely system from Agilent Technologies (Waldbronn, Germany), consisting of G4220A binary pump, G1316C thermostatted column compartment, and G4226A autosampler with G1330B autosampler thermostat coupled to an Agilent 6460 triple quadrupole mass spectrometer equipped with a jet stream electrospray source operating in positive ion mode. The separation was achieved by injecting one1 μL of each sample onto a BEH C18 2.1 × 100 mm, 1.7 μm column (Waters, Milford, MA, United States) held at 50°C in a column oven. The gradient eluents used were H2O 0.1% formic acid (A) and acetonitrile 0.1% formic acid (B) with a flow rate of 500 μL/min. The initial conditions consisted of 0% B, and the following gradient was used with linear increments: 0.54–3.50 min (0.1–9.1% B), 3.50–7.0 (9.1–17.0% B), 7.0–8.0 (17.0–19.70% B), 8.0–8.5 (19.7% B), 8.5–9.0 (19.7–21.2% B), 9.0–10.0 (21.2–59.6% B), 10.0–11.0 (59.6–95.0% B), 11.0–11.5 (95.0% B), 11.5–15.0 (0% B). From 13.0 to 14.8 min, the flow rate was set at 800 μL/min for a faster equilibration of the column. The MS parameters were s optimised for each compound as described in (Supplementary Datasheet S2) information. MRM transitions for the derivatised amino acids were optimised using Mass Hunter MS Optimizer software (Agilent Technologies Inc., Santa Clara, CA, United States). The fragment voltage was set at 380 V, the cell accelerator voltage at 7 V, and the collision energies from 14 to 45 V, nitrogen was used as the collision gas. The data were quantified using MassHunter™ Quantitation software B08.00 (Agilent Technologies Inc., Santa Clara, CA, United States), and the amount of each amino acid was calculated based on the calibration curves.

### Mass Spectrometry Parameters

Jet-stream gas temperature was 290°C with a gas flow of 11 L/min, sheath gas temperature of 325°C, sheath gas flow of 12 L/min. The nebulizer pressure was set to 20 psi, and the capillary voltage was set at 4 kV. The QqQ was run in Dynamic MRM Mode with 2 min retention time windows and 500 msec cycle scans. Data Analysis for amino acid detection-One-way Anova was performed after normalisation of data.

### Radiolabelled Amino Acid Uptake Assay

The Radiolabelled uptake assay was performed as previously described in (83). Brieﬂy, after ten days, PCC cultures in 96 well plates were rinsed with a HEPES-KRH buffer (11 mM d-glucose, pH 7.4) and pre-incubated at 37°C for 5 min. The preincubation buffer was replaced with transport buffer containing 0.2 μM 3[H]-labelled amino acids with a respective amino acid for 50 min at 37°C. A radiolabelled amino acid was used as follows: Aspartic acid (D-Asp 12.9 Ci/mmol), Glutamic acid (L-Gln 50.3 Ci/mmol), Glutamine (L-Glu 51.1 Ci/mmol, L-Leu 56.2 Ci/mmol, and MeAIB 58.7 mCi/mmol prepared in KRH buffer. Cells were lysed with 0.5 N sodium hydroxide (NaOH) for 30 min at 37°C after incubation. The cell lysate was analysed for radioactivity by liquid scintillation counting transferred to scintillation tubes, Minis 2000 (Zins-ser analytic, Frankfurt, Germany) with 2.5 ml Aqua safe 300 plus scintillation liquid (Zinsser analytic). The uptake was measured in counts per minute (cpm) using the liquid scintillation analyser, Tri-Carb 2910 TR (Perkin Elmer, Waltham, MA, United States) (Hellsten et al., 2017). Uptake experiments were performed with *n* = 8 in each group, and further protein quantification was measured by the DC protein assay kit (Pierce, Rockford, IL). Uptake [pmol amino acid/min/mg protein] was calculated using total protein content w.r.t sample. The data illustrate the mean ± SD. The graph was plotted, and the statistical analysis was performed using the software Graph Pad PRISM 5. Unpaired t-tests with 95% conﬁdence interval was performed between WT and KO (*p < 0.05, **p < 0.01, ***p < 0.001).

### Cell Viability

ATP concentrations were measured with the CellTiter-Glo Luminescent Assay (G7570 Promega, United States) using a FLUOstar Omega luminometer (BMG Lab Technologies) according to the manufacturer’s protocol (84). Data were collected from multiple replicate wells for each experiment. In brief...after ten days of culturing and starvation/refeeding experiments performed in 96 well plates with 1 × 104 numbers of PCCs, an equal amount of CellTiter-Glo® Luminescent Cell Viability Assay solution was added according to manufacturer’s instructions (G7570 Promega, United States), and plates were shaken on an orbital shaker for 2 min at 200 rpm and incubated for 10 min. The luminescence was measured using a FLUOstar Omega luminometer (BMG Lab technologies) microplate reader.

### Immunocytochemistry

Cells for Immunocytochemistry (ICC) were washed once with DPBS (Gibco, Invitrogen) and fixed using 4% formaldehyde (Histolab) for 20 min. The cells were then washed 3 × 10 min with DPBS. Immunocytochemistry was performed using antibodies for mTOR (Rabbit mAb #2983, Cell signalling) diluted 1:50 in Super mix blocking solution, LAMP1 (ab24170, Abcam) diluted 1:100, and DAPI (D9542, Sigma Aldrich) diluted in PBS 1:10000. Fluorescent images were taken using the same exposure settings for all pictures using a Zeiss AxioPlan 2 fluorescence microscope, connected to an AxioCamHRm camera (35).

### Image Analysis

For each group, 6–10 pictures were taken. We have used CellProfiler 3.1.9 (85). The analysis was performed to measure co-localisation and intensities of mTOR and LAMP1 protein in PCCs. The CellProfiler pipeline is attached to Supplementary Datasheet S2. In detail, nuclei were first detected in the DAPI image using an Otsu global auto threshold (86). The cytoplasmic area was defined as a ring-shaped area around the nucleus with a width of 100 pixels. The mean intensity of mTOR and Lamp was calculated in the area nuclei + ring-shaped cytoplasm (=“cells”). Co-localisation: mTOR and Lamp signals were first enhanced using a white top-head filter with a feature size of 20 pixels (Enhance or Suppress Features). Co-localisation was only calculated in areas of the filtered image where the intensity was higher than a fixed global threshold to exclude background signal from the analysis. Co-localisation was measured using the Measure Colocalisation of Cell Profiler module, which calculates Pearson’s correlation coefficient. The statistical analysis of the Mean Intensity of Cells (Intensity_MeanIntensity_mTOR_Cells), Lamp (Intensity_MeanIntensity_lamp1 Cells) and the co-localisation (Mean_Cytoplasm_Correlation_Correlation_Lamp_masked_mTOR_masked) was calculated on the distribution of per-image average values of 12–17 images per cell line/condition. Statistical analyses: One-way ANOVA, Tukey’s Multiple Comparison Test were performed, and boxplots, Whiskers (Min to Max) was plotted using Prism 5.

### Phospho-mTOR Antibody Array

Human mTOR signalling Phospho-Specific Antibody Microarray was purchased from Full moon Biosystems. More than 138 site-specific and phospho-specific antibodies for the human mTOR-signalling pathway were immobilised and replicated six times on glass slides. Briefly, cell lysates were prepared using RIPA buffers containing a 1X protease and phosphatase Inhibitor Mini Tablets (Thermo Scientific™ Pierce) and processed according to the manufacturer’s protocol. Then, 30 μg of cell lysates were labelled with 33 μL of biotin. Blocking antibody microarray slides were prepared using a blocking solution for 40 min at room temperature, extensively rinsed with Milli-Q grade water (10 times), and excess water removed. Antibody microarray slides were prepared, together with coupling using protein coupling mix with biotin-labelled cell lysates at room temperature for two hours on an orbital rotating shaker. Antibody array slides were washed four times with 30 ml of 1× Wash Solution; extensive wash was performed ten times using Milli-Q grade water. Bound biotinylated proteins were detected using Cy3-conjugated streptavidin (SA10044, Molecular Probe, R-PE, Lot 1738605). The slides were scanned on a GenePix 4000 scanner, and the images were analysed with GenePix Pro 6.0 (Molecular Devices, Sunnyvale, CA, United States). Ab microarray analysis was carried out following the manufacturer’s protocol using excel macro. In detail, for each spot on the array, median signal intensity was extracted from the array image. For each antibody, the average signal intensity of replicate spots was calculated. The data were labelled as Average Signal Intensity of Replicate Spots. CV of the Replicates means the coefficient of variation for the replicate spots for each antibody. Normalisation was performed for each array slide as follows, determining the median value of the Average Signal Intensity for all antibodies on the array (excluding empty spots, negative controls, and positive markers). This value was represented as Median Signal and further normalised data = Average Signal Intensity of Replicate Spots/Median Signal. The results were labelled as Data Normalised to Median Signal. Using the normalised data, we determined the fold change between WT (wild type) and KO samples. Fold change = KO/WT Sample (Tani et al., 2014).

Further phosphorylation ratio change was computed based on the following equation: the expression of phosphorylated proteins was normalised to corresponding non-phosphorylated protein expression in both WT and KO. Phosphorylated proteins were considered to be differentially expressed when an increase (≥2.0) or decrease (≤0.5) occurred in the ratio of expression levels in KO and among the different treatment groups (Figure 3A; Supplementary Table S5 and Supplementary Datasheet S3).

### Phospho-mTOR Protein Array Analysis

Ingenuity Pathway Analysis (IPA) software (Ingenuity Systems®; www.ingenuity.com) was used to analyse phosphoprotein array data. IPA phosphorylation analysis was performed to determine the metabolic and biological effect of removing SLC38A10 in KO PCCs on the nervous system. Phospho-protein fold changed (FC dataset) was used to determine further and predict the comparative effect on amino acid starvation and refed conditions. From phosphoprotein data, we predicted the list of signalling and metabolic canonical enrich pathways based on z-scores, predicted upstream regulators being significant, disease and biological function as being over-represented, Molecular network potential metabolic interaction in this system, Molecular and biological process predicted to be activated or inhibited extracted and attached in Supplementary Datasheet. The comparison analysis function of IPA was then used to compare all treatments, basal, starved and refed, in which functional clusters differ significantly between the datasets.

### Western Blot

Western blot analysis was performed as previously described according to (Hellsten et al., 2018) along with manufactured antibodies protocol. Briefly, cells were washed in 1XPBS and lysed in 1 ml of RIPA Lysis containing protease and phosphatase inhibitor. Total protein was quantified using the Bradford method (Bio-Rad), separated on 10% Mini-PROTEAN® TGX™ Precast Protein Gels (Bio-Rad, United States). Proteins were blotted to a 0.2 μm PVDF membrane using the Trans-Blot® Turbo™ Mini PVDF Transfer Packs (Bio-Rad, United States) Trans-Blot® Turbo™ Transfer System for 10 min (Bio-Rad, United States) for heavyweight protein and 7 min for low molecular weight protein. Blocking was performed using BSA (Bio-Rad, United States) 5% w/v in PBS with 0.1% Tween 20 (P9416 Merck, Sigma) for one hour at room temperature. Primary antibody blocking was performed according to the manufacturer’s instructions overnight at 4°C. Antibodies used as follows: mTOR (7C10) Rabbit mAb # 2983S (1:1000),Phospho-mTOR (Ser2448) Rabbit mAb #2971S2971S (1:1000), Phospho-4E-BP1 (Thr37/46) (236B4) Rabbit mAb #2855T2855T (1:1000), Phospho-S6 Ribosomal Protein (Ser240/244) (2F9) Rabbit mAb # 4856S4856S (1:1000), 4E-BP1 #9452S9452S (1:1000), PhosphoPhospho-p70 S6 Kinase (Thr389) (108D2) Rabbit mAb # 9234T9234T (1:1000), eIF4B # 9742S (1:1000), (all from Cell signalling), Anti--β-Actin antibody, Mouse monoclonal # A1978A1978A1978 (Sigma). After overnight blocking, secondary Anti-rabbit IgG, HRP-linked Antibody #7074, diluted to 1:5000 (cell Signalling), was the solution used for one hour at room temperature, and the HRP goat *α*-mouse, diluted 1:10000 (Invitrogen). The membrane was developed using Clarity Western ECL Substrate (Bio-Rad) and visualised using a CCD camera (ChemiDoc, Bio-Rad). Blots were further analysed was using Fuji software. A graph was plotted after normalised beta-actin data. Densitometry analysis was performed using ImageJ (Fuji) (Hellsten et al., 2018). Statistical analysis, *t*-test, was performed, and an additional graph was plotted using Prism 5.

### Metabolomics Analysis

Metabolic profiling by GC-MS was performed at the Swedish Metabolomics Centre in Umeå, Sweden. Information about reagents, solvents, standards, and stable isotopes internal standards can be found as Supplementary Material. Sample Preparation: Extraction was performed as previously described (Jiye et al., 2005). 350 µL extraction buffer (80/20 v/v methanol: water), including internal standards, and a tungsten bead was added to the cell samples. The samples were shaken at 30 Hz for 2 min in a mixer mill. The samples were centrifuged at +4°C, 14,000 rpm, for 10 min 250 µL of the supernatant was transferred to micro vials, and solvents were evaporated to dryness.

GCMS Analysis: Derivatisation and GCMS analyses were performed as described previously (Jiye et al., 2005; Schauer et al., 2005) except that the samples were derivatised in a final volume of 30 µL instead of 90 µL. One μL of the derivatised samples was injected in splitless mode by an L-PAL3 autosampler (CTC Analytics AG, Switzerland) into an Agilent 7890B gas chromatograph equipped with a 10 m × 0.18 mm fused silica capillary column with a chemically bonded 0.18 μm Rxi-5 Sil MS stationary phase (Restek Corporation, US) The injector temperature was 270°C, the purge flow rate was 20 ml min-1, and the purge was turned on after 60 s. The gas flow rate through the column was 1 ml min-1, the column temperature was held at 70°C for 2 min, then increased by 40°C min-1–320°C and held there for 2 min. The column effluent was introduced into the ion source of a Pegasus BT time-of-flight mass spectrometer, GC/TOFMS (Leco Corp., St Joseph, MI, United States). The transfer line and the ion source temperatures were 250 and 200°C, respectively. Ions were generated by a 70-eV electron beam at an ionised ionisation current of 2.0 mA, and 30 spectra s-1 were recorded in the mass range m/z 50–800. The acceleration voltage was turned on after a solvent delay of 150 s. The detector voltage was 1800–2300 V.

### Statistic Data Analysis

For the GC-MS data, all non-processed MS-files from the metabolic analysis were exported from the ChromaTOF software in NetCDF format to MATLAB® R2016a (Mathworks, Natick, MA, United States), where all data pre-treatment procedures, such as base-line correction, chromatogram alignment, data compression, and Multivariate Curve Resolution were performed using custom scripts. The extracted mass spectra were identified by comparisons of their retention index and mass spectra with libraries of retention time indices and mass spectra (Deitmer et al., 2003). Mass spectra and retention index comparison was performed using NIST MS 2.0 software. Fold change was calculated from data from the Umea Metabolomics centre, two-way ANOVA was performed, and the bar graph was plotted using Prism 5.

### RNA Isolation and Transcriptome Analysis

Primary cortex cortical cultures of WT and KO (*n* = 4) were used for RNA extraction. According to the manufacturer’s instructions, the RNA was isolated using an Allprep DNA/RNA micro kit (Qiagen). Samples were stored at −80°C until samples were given for sequencing at Uppsala NGI. The libraries were prepared for sequencing on the Ion Chef System and sequenced on the Ion S5 XL on 540 chips using IonCode adapters. Sequencing libraries for 32 individual samples were prepared from 180 to 280 ng total RNA using the Ion AmpliSeq™ Transcriptome Mouse Gene Expression Kit (Cat. Nos. A36553, A36554, and A36555, Thermo Fischer Scientific) according to the manufacturer’s protocol. The SNP&SEQ Technology Platform in Uppsala then performed sequencing on Ion S5 XL on a 540 chips machine, and data from three lanes of sequencing were uploaded on Uppmax.

### Network and Pathway Analysis

Gene list was processed, as mentioned in Supplementary Datasheet S1. Further, data were subjected to pathway analysis. PA analysis was performed by setting Gene only, specific cell selection in Cell- Astrocyte, Neurons, Stem Cell, Brain-Cerebral cortex, and CNS cell lines. The data set used fold change (FC- KO *Vs.* WT) (SE1- Ampliseq gene list), selected as an expression ratio which is further converted into fold change. Cut off was set −2 and 2 and *p*-value 0.05. For disease and function Amino acid, carbohydrate, cell cycle death, cell morphology, cell signalling, gene expression, lipid metabolism, metabolic disease, molecular transport, Nervous system, Neurological disease, post-translation modification, vitamin-mineral metabolism. List of changes SLCs was extracted from gene list and further analysed using IPA analysis with sample parameters. IPA analysis was performed by setting Gene only, specific cells selection in Cell- Astrocyte, neurons, stem Cell, brain-cerebral cortex, and CNS cell lines. The data set used fold change (SE1) for pathway analysis, and the cut-off was set −2 ≥≤ 2 and *p*-value 0.05. For disease and function amino acid, carbohydrate, cell cycle death, cell morphology, cell signalling, gene expression, lipid metabolism, metabolic disease, molecular transport, nervous system, neurological disease, post-translation modification, vitamin-mineral metabolism. IPA analysis was performed by setting Gene only, specific cells selection in Cell- Astrocyte, Neurons, Stem Cell, Brain-Cerebral cortex, and CNS cell lines. Genesis versions 1.8 9) and HemI_1.0_alpha (10) were used to generate heat maps used in figures. “AmpliSeq data have been deposited in the Array Express database at EMBL-EBI (www.ebi.ac.uk/array express) under accession number E-MTAB-10413Animals.

## Data Availability

The datasets presented in this study can be found in online repositories. The names of the repository/repositories and accession number(s) can be found below: Array Express database at EMBL-EBI (www.ebi.ac.uk/array express) under accession number E-MTAB-10413.
