# Supplementary material for: SLC38A10 Regulate Glutamate Homeostasis and Modulate the AKT/TSC2/mTOR Pathway in Mouse Primary Cortex Cells
Source: Front Cell Dev Biol. 2022 Apr 5;10:854397. doi: 10.3389/fcell.2022.854397 (PMC9017388; doi:10.3389/fcell.2022.854397)
Supplement: Supplementary file 1 [file DataSheet2.PDF]

Supplementary Data S2- Cell profiler pipeline used for analysis for Colocalization mTOR and LAMP1 protein in PCCs.

SLC38A10 Regulate Glutamate Homeostasis and modulate the AKT/TSC2/mTOR Pathway in mouse cortex cells

CellProfiler data: plotting
Project: Rekha Tripathi - mTOR, Lamp

Data: 20200320_mTOR_LAMP_BIOVIS_2020_AT_63X

- Ch1: DAPI
- Ch2: mTOR: protein of interest
- Ch3: Lamp: lysosome marker

- Conditions: Basal, starved, refeed
-
- CP Pipeline: 2020421_imageInt_correlation.cpproj

2020-04-21 Anna Klemm, BioImage Informatics Facility, SciLifeLab, anna.klemm@it.uu.se

Importing packages and data

In [1]:
```
import pandas as pd import re
import seaborn as sns
import matplotlib.pyplot as
plt
```

In [2]:
```
path_cpout = r"C:\Users\Anna\Documents\projects\Rekha_Tripathi\20200421_Imagei
ntensity_correlation_higherThres"
image = pd.read_csv(path_cpout + "\MyExpt_image.csv") nuclei =
pd.read_csv(path_cpout + "\MyExpt_nuclei.csv") cytoplasm =
pd.read_csv(path_cpout + "\MyExpt_Cytoplasm.csv")
```

In [3]:
```
#list(image.columns)
```

In [4]:
```
image['average_areaPerCell_mTOR'] = image['AreaOccupied_AreaOccupied_mTOR_mask
ed_Cytoplasm']/image['Count_Cytoplasm']
image['average_areaPerCell_lamp'] = image['AreaOccupied_AreaOccupied_Lamp_mask
ed_Cytoplasm']/image['Count_Cytoplasm']
image['average_areaPerCellDonut_mTOR'] = image['AreaOccupied_AreaOccupied_mTOR
_masked_Cytoplasm_donut']/image['Count_Cytoplasm']
```

mTOR: mean intensity image within cytoplasm (Propagation, manual)
The level of mTOR stays the same under starvation in the KOKO mutant

In [5]:
```
sns.boxplot(x='Metadata_Treatment', y= 'Intensity_MeanIntensity_mtor_Cytoplas
m',  order=["Cont", "starv", "Refed"], hue= 'Metadata_CellLine',
                    data=image, palette="Set2")
ax = sns.swarmplot(x='Metadata_Treatment', y= 'Intensity_MeanIntensity_mtor_Cy
toplasm',  order=["Cont", "starv", "Refed"], hue= 'Metadata_CellLine',
                    data=image, color=".25", dodge=True)
```

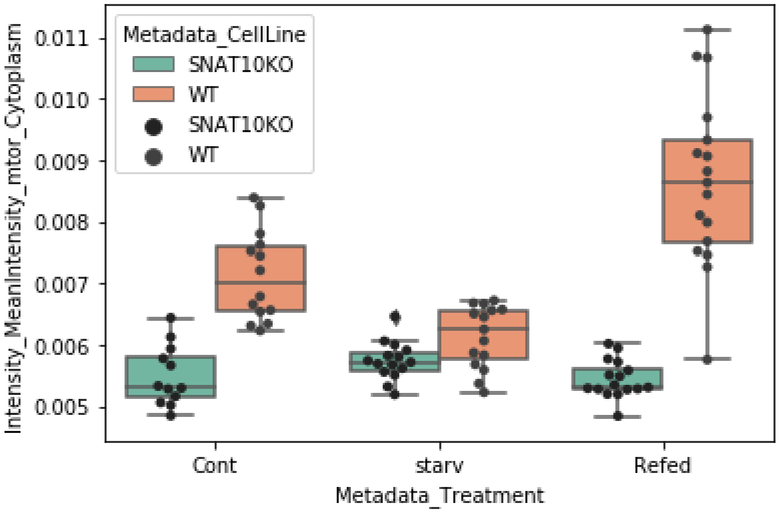

control: mTOR: mean intensity image within "donut" cytoplasm (Distance-N, 100px)

In [6]:
```
sns.boxplot(x='Metadata_Treatment', y= 'Intensity_MeanIntensity_mtor_Cytoplasm
_donut',  order=["Cont", "starv", "Refed"], hue= 'Metadata_CellLine',
                    data=image, palette="Set2")
ax = sns.swarmplot(x='Metadata_Treatment', y= 'Intensity_MeanIntensity_mtor_Cy
toplasm_donut',  order=["Cont", "starv", "Refed"], hue= 'Metadata_CellLine',
                    data=image, color=".25", dodge=True)
```

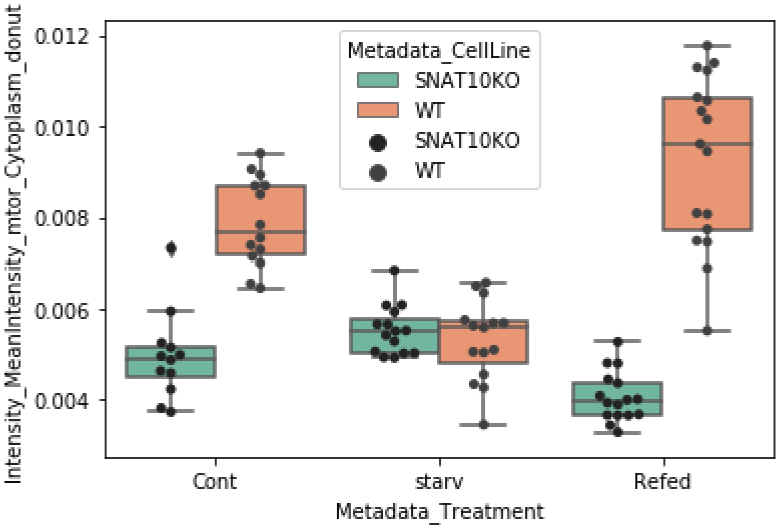

mTOR-area over threshold, cytoplasm "donut-cells"

KOKO has less aggregates under starved condition, recovery when refed. WT is always in aggregates

In [16]:
```
sns.boxplot(x="Metadata_Treatment", y='average_areaPerCellDonut_mtor',  order=
["Cont", "starv", "Refed"], hue= 'Metadata_CellLine',
                        data=image, palette="Set2")
ax = sns.swarmplot(x='Metadata_Treatment', y= 'average_areaPerCellDonut_mtor',
order=["Cont", "starv", "Refed"], hue= 'Metadata_CellLine',
                        data=image, color=".25", dodge=True)
```

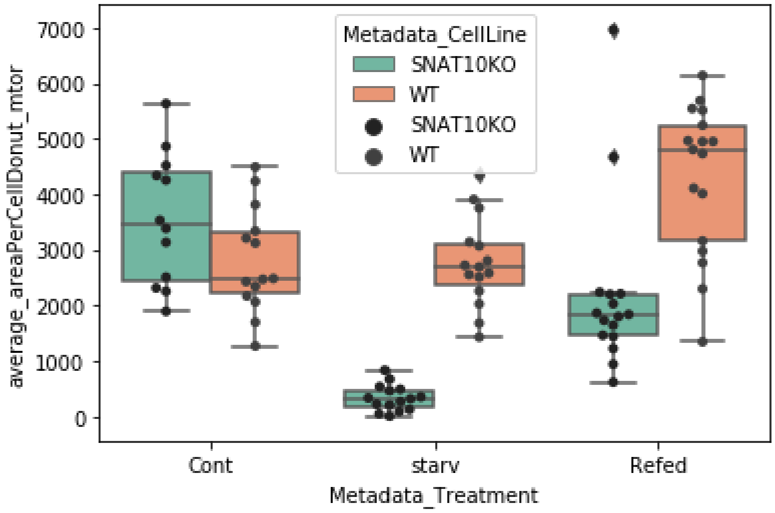

Co-localisation correlation

Pearson

SNATKO: SNATKO starved: few mTOR aggregates do not overlap with lamp; SNATKO refed: fewer objects that exist overlapping with lamp WT. mTOR aggregates always overlap with lamp.

In [8]:
```
sns.boxplot(x="Metadata_Treatment", y='Mean_Cytoplasm_Correlation_Correlation_
Lamp_masked_mTor_masked',  order=["Cont", "starv", "Refed"], hue= 'Metadata_Ce
llLine',
                    data=image, palette="Set2")
ax = sns.swarmplot(x='Metadata_Treatment', y= 'Mean_Cytoplasm_Correlation_Corr
elation_Lamp_masked_mTor_masked',  order=["Cont", "starv", "Refed"], hue= 'Met
adata_CellLine',
                    data=image, color=".25", dodge=True)
```

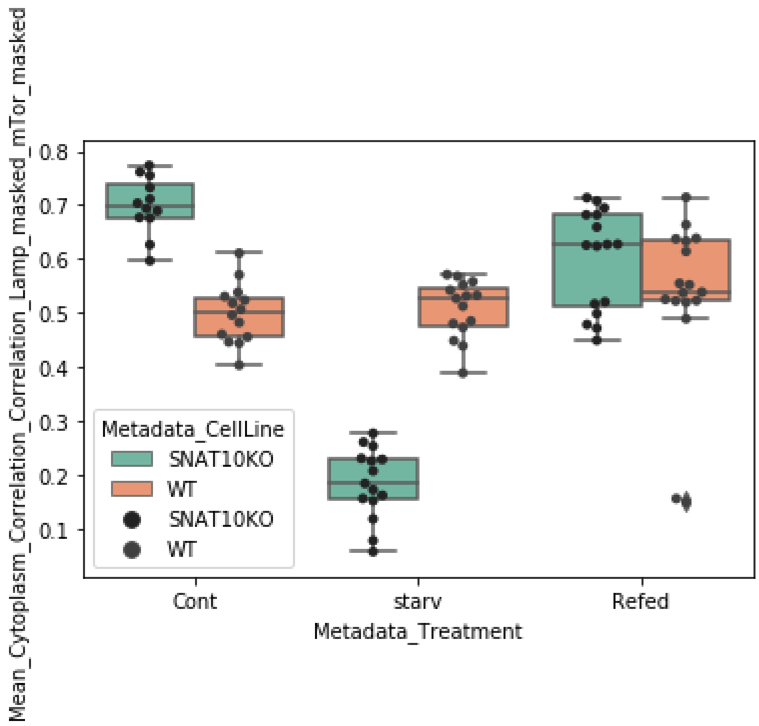

Manders M1

In [12]:
```
sns.boxplot(x="Metadata_Treatment", y='Mean_Cytoplasm_Correlation_Manders_Lamp
_masked_mTor_masked',  order=["Cont", "starv", "Refed"], hue= 'Metadata_CellLi
ne',
                    data=image, palette="Set2")
ax = sns.swarmplot(x='Metadata_Treatment', y= 'Mean_Cytoplasm_Correlation_Mand
ers_Lamp_masked_mTor_masked',  order=["Cont", "starv", "Refed"], hue= 'Metadat
a_CellLine',
                    data=image, color=".25", dodge=True)
# 'Mean_Cytoplasm_Correlation_Manders_mTor_masked_Lamp_masked',
```

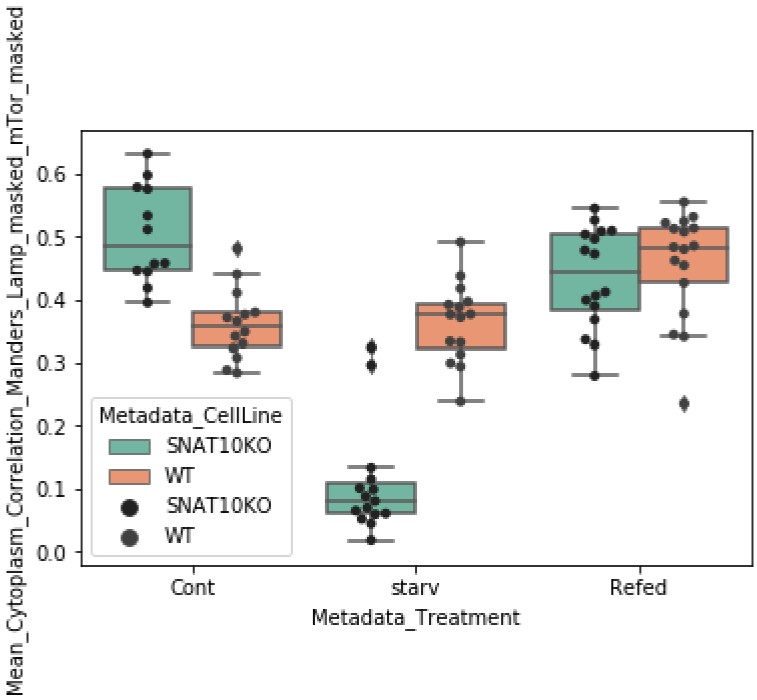

Manders M2

In [13]:
```
sns.boxplot(x="Metadata_Treatment", y='Mean_Cytoplasm_Correlation_Manders_mTor_masked_Lamp_masked',  order=["Cont", "starv", "Refed"], hue= 'Metadata_CellLine',
                    data=image, palette="Set2")
ax = sns.swarmplot(x='Metadata_Treatment', y= 'Mean_Cytoplasm_Correlation_Manders_mTor_masked_Lamp_masked',  order=["Cont", "starv", "Refed"], hue= 'Metadata_CellLine',
                    data=image, color=".25", dodge=True)
# 'Mean_Cytoplasm_Correlation_Manders_mTor_masked_Lamp_masked'
```

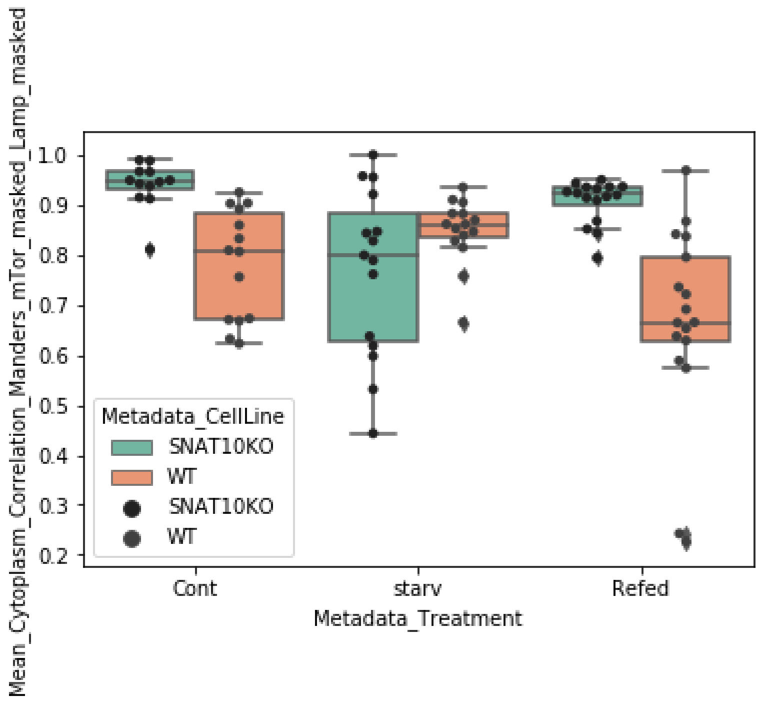

Lamp: mean intensity within cells

In [14]:
```
ax = sns.boxplot(x='Metadata_Treatment', y= 'Intensity_MeanIntensity_Lamp_Cell
s',  order=["Cont", "starv", "Refed"], hue= 'Metadata_CellLine',
                    data=image, palette="Set2")
ax = sns.swarmplot(x='Metadata_Treatment', y= 'Intensity_MeanIntensity_Lamp_Ce
lls',  order=["Cont", "starv", "Refed"], hue= 'Metadata_CellLine',
                    data=image, color=".25", dodge=True)
```

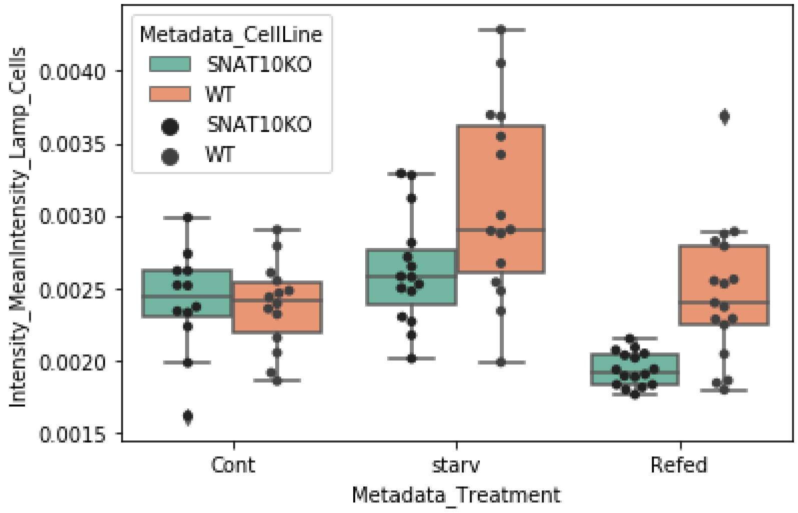

In [ ]:
```
```
