# Supplementary material for: SLC38A10 Regulate Glutamate Homeostasis and Modulate the AKT/TSC2/mTOR Pathway in Mouse Primary Cortex Cells
Source: Front Cell Dev Biol. 2022 Apr 5;10:854397. doi: 10.3389/fcell.2022.854397 (PMC9017388; doi:10.3389/fcell.2022.854397)
Supplement: Supplementary file 3 [file DataSheet4.PDF]

Supplementary Data-Tables

SLC38A10 Regulate Glutamate Homeostasis and modulate the AKT/TSC2/mTOR Pathway in mouse cortex cells

Table 1 Upstream regulator detected in KO PCCs at Basal condition by using AmpliSeq gene list

Upstream Regulator	Expr Log Ratio	Molecule Type	Predicted Activation State	Activation z-score	p-value of overlap	Target Molecules in Dataset
FEV		TR	Inhibited	-2.449	0.0167	ANXA2,FOS,JAM2,LPAR1,VIM,VTN
STAT1		TR	Inhibited	-2.219	0.000955	CXCL10,GBP2,ICAM1,IFI16,IRF8,Oasl2
SOX2	-0.45	TR	Inhibited	-2.72	0.000804	CASR,CLDN11,COL11A2,CTNNAL1,GJC3,ID3,LPAR1,MOBP,PLP1,PROM1,TSPAN15
CSF1	-3.08	c	Inhibited	-2.354	1.08E-11	CSF1,CTSD,CTSH,EGR2,GPNMB,GPR34,HLA-A,ITGAM,PTPRC,SPARC,SPI1,SPP1,TGFB1
MKNK1	-0.144	K	Inhibited	-3	0.000679	ANXA2,ANXA5,CP,FOS,FOSB,GPC4,PLTP,SPARC,VIM
CREB1	0.097	TR	Inhibited	-2.159	0.000244	ARC,ATF3,EGR1,EGR2,FOS,FOSB,MNS1,VEGFA
SREBF1	-0.385	TR	Inhibited	-2.449	0.00135	C5AR1,CD14,GPNMB,HFE,PLEKHA4,TNFRSF1B
TNF		C	Inhibited	-3.255	9.63E-07	ADAM8,CD14,CD44,CSF1,CXCL10,CXCL16,FAS,ICAM1,LGALS9B,PLAU,VIM
HTT	0.057	TR	Inhibited	-3.207	0.000065	COL18A1,COL4A1,CRYAB,CSF1R,DCN,EGR1,EGR2,EMP3,FOS,IGF2,MGP,MMP2,PRELP,PROM1,PSMB8,SERPINF1,SERPING1,SLC40A1,SPP1,TGM2,THBS2
APEX1	-0.291	E	Inhibited	-2.219	1.17E-05	EGR1,EGR2,FOS,FOSB,NPAS4
IFNG		C	Inhibited	-3.94	1.63E-11	CASP8,CD14,CD68,CSF1,CXCL10,CXCL16,EGR2,FAM107A,FAS,FOSB,GBP2,GJB2,GPER1,ICAM1,IFI16,IRF5,IRF8,LIF,LOX,P2RY6,PLAU,S1PR3,SOD3,TGFBR2,TLR4,VEGFA
IL1B		C	Inhibited	-2.367	0.000542	CSF1,CXCL10,FAS,FOS,ICAM1,PLAU,VIM
QKI	-0.605	other	Inhibited	-2.449	9.63E-05	ATP6V0D2,CD36,CNN1,CTSS,ITGAM,VIM
TSC2	0.3	other	Activated	3.771	5.24E-15	A2M,ANXA1,ANXA2,ARC,ATF3,CP,CRYAB,CXCL10,EMP3,FAM107A,FOS,GPNMB,LGALS3,MGP,PRELP,RASSF4,UCP2,VAMP8
ADORA2A	0.799	GPCR	Activated	2	0.164	CSF1R,EGR2,FOS,SPARC
PTGER4	0.422	GPCR	Activated	2.035	0.000487	CP,Ctla2a/Ctla2b,CXCL10,CYBB,GBP2,IFI16,Irgm1,NCF1,NCF2,TLR8
Irgm1	-2.491	other	Activated	2.169	0.00816	CXCL10,CXCL16,GBP2,IFI16,Oasl2

Transcription regulator (TR), cytokine-Kinase-K- Enzyme- E, G-protein coupled receptor-GPCR

Table 2 Metabolic Disease predicted because of an absence of SLC38A10 gene in KO PCCs

Categories	Diseases or Functions Annotation	p-value	Molecules	# Molecules
Metabolic disease, organismal Injury and Abnormalities	Amyloidosis	3.69E-04	ACTA2,BMP6,CAST,CD14,CD36,CD68,CNN1,CTSD,ICAM1,PAWR,PLTP,PRDX6,TGFB1,TGFBR2,TTR,VIM	16
Metabolic Disease, Neurological Disease, Organismal Injury and Abnormalities, Psychological Disorders	Alzheimer's disease	2.26E-03	ACTA2,BMP6,CD14,CD36,CD68,CNN1,CTSD,ICAM1,PAWR,PLTP,PRDX6,TGFB1,TGFBR2,TTR	14
Metabolic Disease, Neurological Disease, Organismal Injury and Abnormalities	Beta amyloidosis	2.05E-02	CAST,VIM	2
Cardiovascular Disease, Metabolic Disease, Neurological Disease, Organismal Injury and Abnormalities, Psychological Disorders	Cerebral amyloid angiopathy	3.04E-02	ACTA2,CD36	2

Table 3 Genes in the Neurodegeneration network

Gene fold change value affected in KO PPCs involved in neurodegenerative disease analysed by IPA software.

Genes in the Neurodegeneration network	Basal FC	B27 starved FC	AA starved FC	AA Refeed FC
SLC1A2	-0.361712143	2.316464	1.283141	1.713699
SLC7A11	-0.512543891	2.469864	-1.38103	0.517474
SLC7A8	0.987558745	0.938724	0.848879	0.248764
SLC1A3	-0.533132817	1.083406	-0.15136	0.544938
SLC18A2	-0.578096682	0.557634	0.603711	0.316997
SLC9A1	-0.046727498	0.668414	0.274336	0.508396
SLC4A7	-0.422981918	-0.02757	0.086138	-0.54122
SLC33A1	-0.099056625	-0.01459	-0.02435	0.138467
SLC19A2	-0.103163736	-0.49233	-0.68607	-0.67357
SLC12A7	-2.121435831	-0.7803	-1.9092	-0.54207

Table 4 AmpliSeq fold changed expression in KO cells members of the SLC38 family

Gene	Basal	B27 Starved	Starved	Refeed
Slc38a1	0.299364	0.451219	0.144581	0.535975
Slc38a2	-0.18904	-0.08955	-1.1466	0.441899
Slc38a3	-0.7232	-0.11199	-0.82898	0.932454
Slc38a6	-0.06326	-0.01575	0.126049	0.141985
Slc38a7	0.462057	0.030346	-0.1067	-0.81729
Slc38a9	0.051023	0.458897	0.128587	-0.23243
Slc38a10	-6.95659	-5.59749	-6.2049	-5.54299
Slc38a11	-1.03571	-1.71361	-1.22073	-0.58963

Table 5 List of phospho mTOR protein detected using Phospho Explorer Array. Data represented in fold change KO against WT

Protein name	Control	Starved	Refeed
4E-BP1 (P-Ser65)/4E-BP1	2.068300116	8.740601177	2.033872453
4E-BP1 (Phospho-Thr36) /4E-BP1	0.995274025	0.69784196	0.910860499
4E-BP1 (Phospho-Thr45) /4E-BP1	1.158023339	0.576934273	0.961756136
4E-BP1 (Phospho-Thr70)/4E-BP1	1.69124967	6.945637749	2.235757394
PFKFB2) (Phospho-Ser483) /PFKFB2	0.749355978	0.257190847	1.095789659

AKT (Phospho-Ser473) /AKT	1.006293491	0.67374437	2.457160578
AKT (Phospho-Thr308) /AKT	0.82835038	0.592135019	1.5841635
AKT (Phospho-Tyr326)/AKT	0.902564084	0.756984396	0.938533751
AKT1 (Phospho-Ser124)/AKT1	1.819414421	2.277930243	2.128461445
AKT1 (Phospho-Ser246)/AKT1	0.697613326	1.500330249	0.33817443
AKT1 (Phospho-Thr450)/AKT1	1.422142863	3.666683684	1.910430087
AKT1 (Phospho-Thr72)/AKT1	0.7981878	1.581998308	0.7592732
AKT1 (Phospho-Tyr474)/AKT1	0.604169459	0.875881581	0.282234702
AKT1S1 (Phospho-Thr246)/AKT1S1	2.184951781	6.820615054	1.772700725
AKT2 (Phospho-Ser474) /AKT2	1.011831679	0.900249982	1.129392409
AMPK1 (Phospho-Thr174)/AMPK1	1.288441318	0.60920378	0.676997462
AMPK1/AMPK2 (Phospho-Ser485/491) /AMPK1/AMPK2	1.11968715	1.162435361	2.839235751
AMPKbeta1 (Phospho-Ser182)/AMPKbeta1	1.802062954	3.137342711	1.495795075
BAD (Phospho-Ser112) /BAD	1.094736344	1.563667624	0.938069526
BAD (Phospho-Ser134)/BAD	1.22976858	1.175162991	3.359474475
BAD (Phospho-Ser136) /BAD	0.993941049	1.709616395	0.977282073
BAD (Phospho-Ser155) /BAD	1.026392932	1.066773194	1.199652318
BAD (Phospho-Ser91/128)/BAD	1.479726443	0.79938235	2.451197578
eIF2 alpha (Phospho-Ser51) /eIF2 alpha	1.128259538	0.39664224	1.457860074
eIF4E (Phospho-Ser209) /eIF4E	1.042950547	0.900886237	0.941678854
eIF4G (Phospho-Ser1108)/eIF4G	0.815128243	1.665951997	0.765403891
ERK1-p44/42 MAP Kinase (Phospho-Thr202) /ERK1-p44/42 MAP Kinase	0.971546738	0.897337002	0.676036745
ERK1-p44/42 MAP Kinase (Phospho-Tyr204) /ERK1-p44/42 MAP Kinase	0.991972691	1.077205929	0.907105233
ERK3 (Phospho-Ser189)/ERK3	1.034719696	2.570912972	1.301004503

GSK3 alpha (Phospho-Ser21) /GSK3 alpha	0.745863171	0.37862076	2.384256023
GSK3 beta (Phospho-Ser9) /GSK3 alpha	0.815672404	0.59739187	1.493271267
GSK3a-b (Phospho-Tyr216/279)/GSK3a-b	1.196805341	6.016119084	1.856294773
mTOR (Phospho-Ser2448)	1.096749111	0.676672061	1.113437368
mTOR (Phospho-Ser2481)	0.720731786	0.810980964	1.279742671
mTOR (Phospho-Thr2446)	0.910028201	4.464993408	0.815542839
P70S6K (Phospho-Ser371)/P70S6K	1.551239514	4.666890267	0.91146151
P70S6K (Phospho-Ser411) /P70S6K	1.319607427	1.405743434	1.085007338
P70S6K (Phospho-Ser418)/P70S6K	1.576401705	3.203412558	0.530775129
P70S6K (Phospho-Ser424) /P70S6K	1.128704114	0.484126696	0.706751815
P70S6K (Phospho-Thr229)/P70S6K	1.479909416	5.811784424	1.226907651
P70S6K (Phospho-Thr421)	1.210177571	3.377626695	1.826795537
P70S6k-beta (Phospho-Ser423)/P70S6k-beta	0.942007836	1.605653865	1.883340423
P90RSK (Phospho-Ser380)/P90RSK	1.02086063	2.303481498	0.427726364
P90RSK (Phospho-Thr359/Ser363)/P90RSK	2.124071246	4.804978232	2.311095079
P90RSK (Phospho-Thr573)/P90RSK	1.063059527	2.42744666	0.912104173
PDK1 (Phospho-Ser241) /PDK1	1.359144892	0.403432913	1.564496037
PI3-kinase p85-subunit alpha/gamma (Phospho-Tyr467/Tyr199)	1.005004408	3.598760529	0.825077193
PKC alpha (Phospho-Tyr657)/PKC alpha	0.798083574	0.367202195	0.93860931
PKC alpha/beta II (Phospho-Thr638)	0.699886732	0.449222887	1.050317321
PPAR-b (Phospho-Thr1457)/PPAR-b	0.825254325	2.568355467	1.248954848
PPAR-r (Phospho-Ser112)/PPAR-r	1.120269311	3.775391692	1.073290067
PTEN (Phospho-Ser370) /PTEN	0.850041561	0.462092522	0.689767091
PTEN (Phospho-Ser380)/PTEN	0.775435191	0.816496745	3.131296163
PTEN (Phospho-Ser380/Thr382/Thr383) /PTEN	0.955550719	0.80871247	1.166333231

Rho/Rac guanine nucleotide exchange factor 2 (Phospho-Ser885)	0.890668583	1.793329162	1.177283963
TSC2 (Phospho-Ser939)/Tuberin	0.797667608	0.967082018	1.817536409
TSC2 (Phospho-Thr1462)/Tuberin	0.771387317	1.625514363	0.453722062
