# Supplementary material for: SLC38A10 Regulate Glutamate Homeostasis and Modulate the AKT/TSC2/mTOR Pathway in Mouse Primary Cortex Cells
Source: Front Cell Dev Biol. 2022 Apr 5;10:854397. doi: 10.3389/fcell.2022.854397 (PMC9017388; doi:10.3389/fcell.2022.854397)
Supplement: Supplementary file 5 [file DataSheet3.PDF]

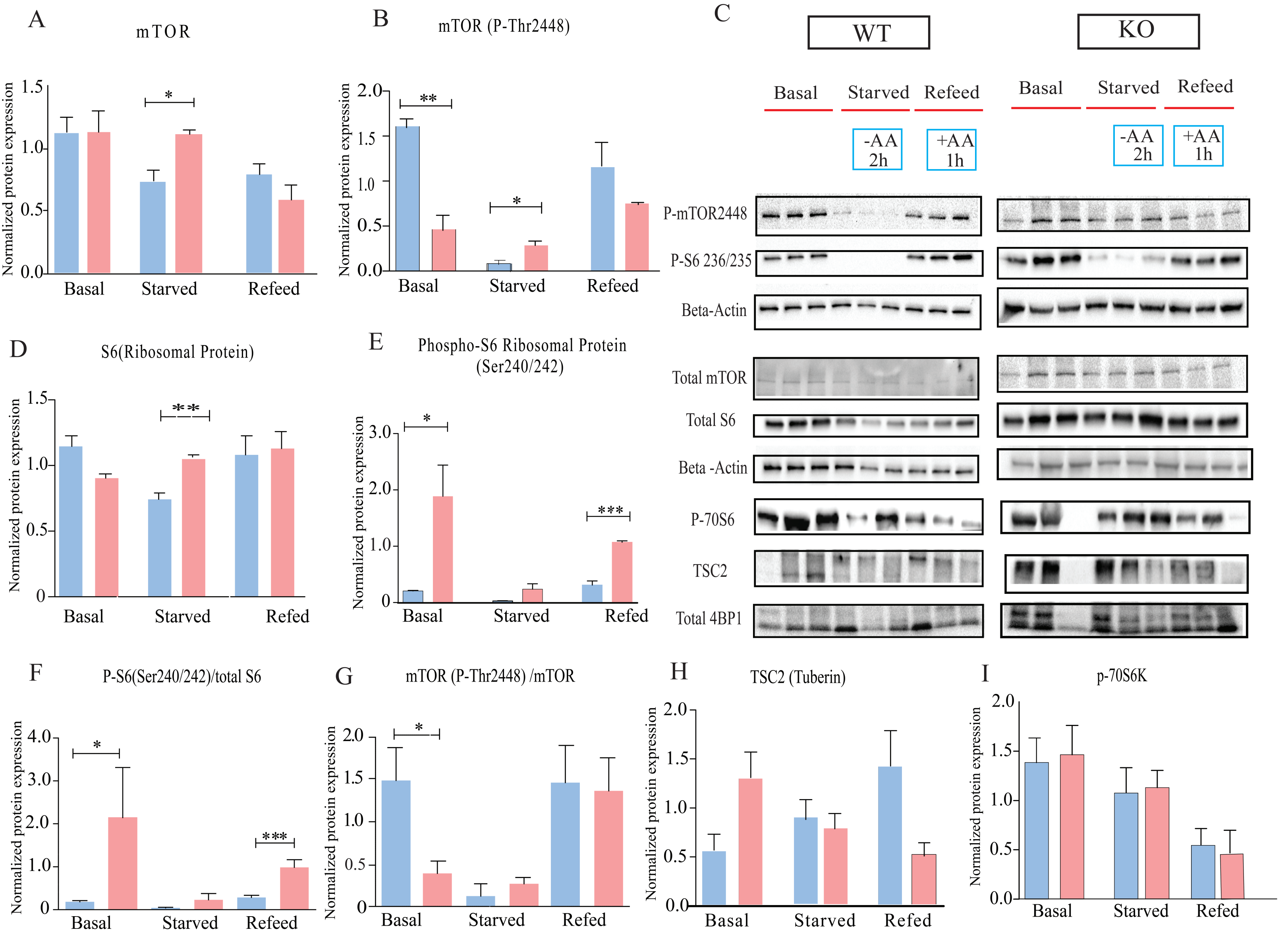

Supplementary Figure 2 (SF2) (A). Immunoblot represents total mTOR, p mTOR2448, Total S6, P-S6 (240/242), p70S6, and total TSC2. (I- K) Quantification of Immunoblot data from total lysates of WT and KO PCCs, for (I) total mTOR, (J) mTOR (P-Thr2448), and (K) the ratio of the two, with the indicated antibodies. (L) Quantification of Immunoblot data from total lysates of WT and KO PCCs for total TSC2. (M-O) Quantification of Immunoblot data from total lysates of WT and KO PCCs for (M) totalS6 and (N) S6(P-Ser240/242) and (O) The ratio of the two, with indicated antibodies. (P) Quantification of Immunoblot data from total lysates of WT and KO PCCs for total p70S6K.
