# Supplementary material for: SLC38A10 Regulate Glutamate Homeostasis and Modulate the AKT/TSC2/mTOR Pathway in Mouse Primary Cortex Cells
Source: Front Cell Dev Biol. 2022 Apr 5;10:854397. doi: 10.3389/fcell.2022.854397 (PMC9017388; doi:10.3389/fcell.2022.854397)
Supplement: Supplementary file 6 [file DataSheet1.PDF]

Supplementary Data S1

Transcriptomic

SLC38A10 Regulate Glutamate Homeostasis and modulate the AKT/TSC2/mTOR Pathway in mouse cortex cells

AmpliSeq analysis was performed using a National Genomics Infrastructure (NGI), SciLifeLab platform, Uppsala.

Pre-processing data was performed as follows-

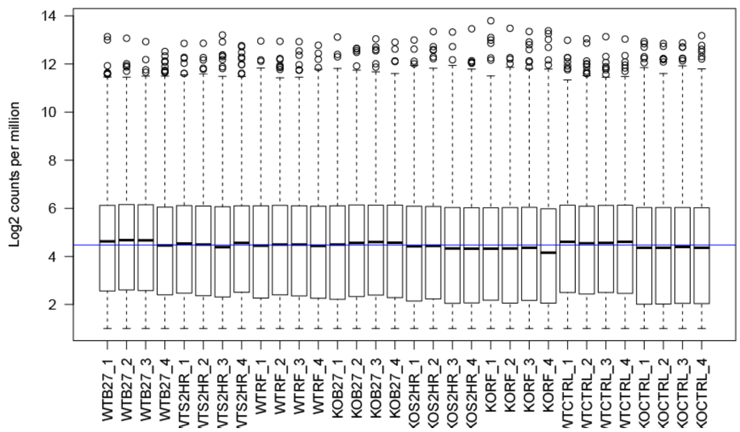

Figures 1. **Boxplot showing data after normalisation for uneven sequencing depth and removal of slow expressed genes. Abbreviations; WTB27: Wild type starved of B27; WTS2HR: Wild type starved for 2HR; WTRF: Wild type refeed after 2h starvation; WTCTRL: Wild type cultured under normal (Basal) conditions; KOB27:KO starved of B27; KOS2HR:KO starved for 2HR; KORF:KO refeed after 2h starvation; KO CTRL:KO cultured under basal conditions**

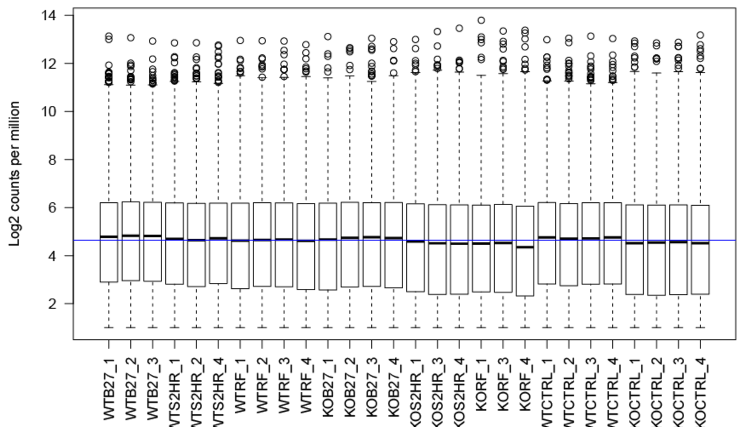

Figure 2. **Box plot showing data after normalisation for uneven sequencing depth and removal of low expressed genes. Based on the clustering in Supplementary Figure 3, four samples: WTB27_4, WTS2HR_3, KOS2HR_2, KORF_2, were**

removed. Abbreviations; WTB27: Wild type starved of B27; WTS2HR: Wild type starved for 2HR; WTRF: Wild type refed after 2h starvation; WTCTRL: Wild type cultured under normal conditions; KOB27:KO starved of B27; KOS2HR:KO starved for 2HR; KORF:KO refeed after 2h starvation; KO CTRL:KO PCCs basal conditions

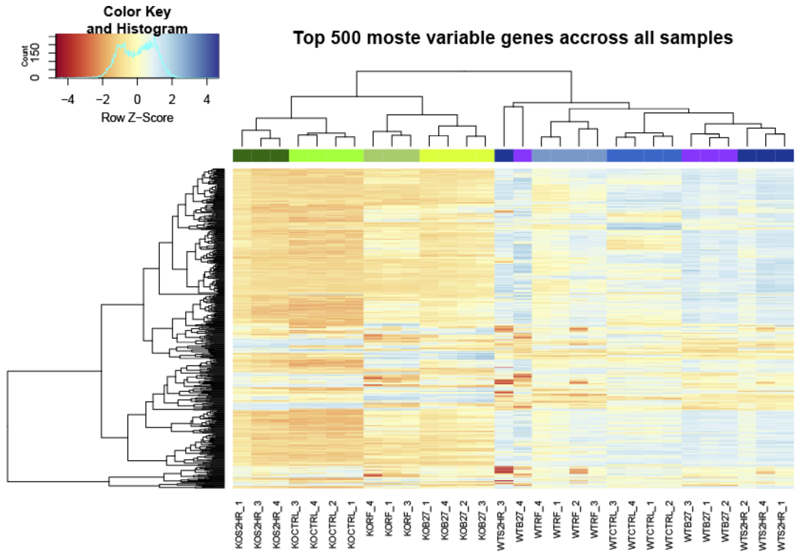

Figure 3. **Heat map over the top 500 most differential expressed genes. Dendograms show clustering by sample, column wise and by gene row wise.**

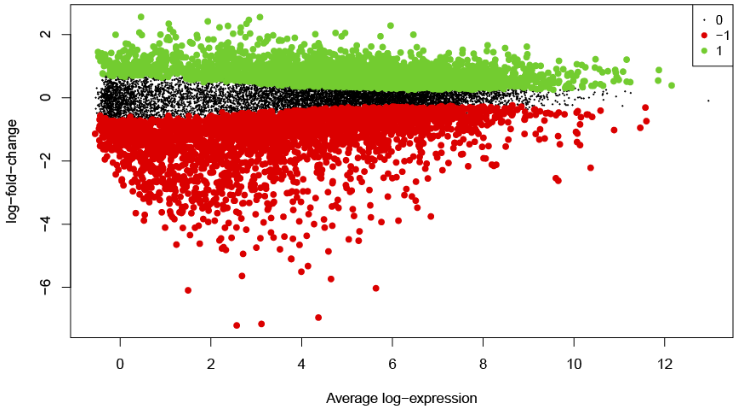

Figure 4. **MDB plot showing differential expression of all genes between KO and WT basal groups.**

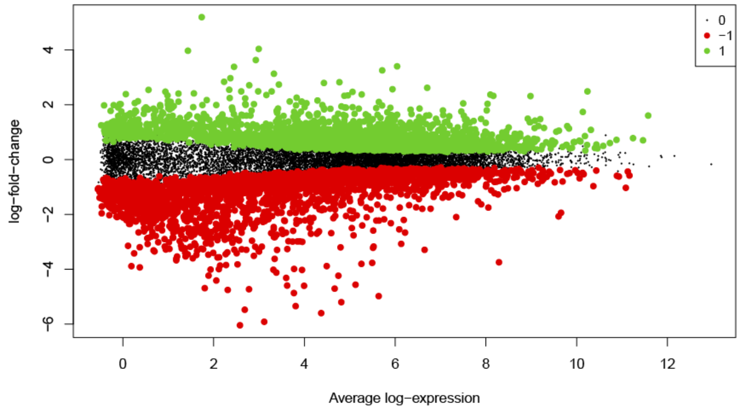

Figure 5. ***MDB plot showing differential expression of all genes between KO and WT B27 starvation groups.***

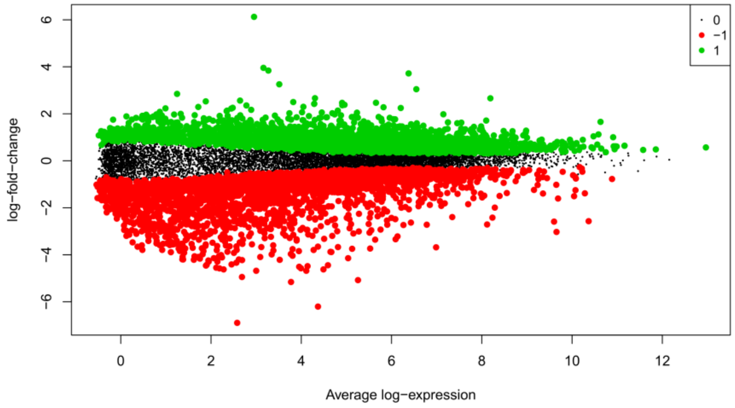

***Figure 6*. MDB plot showing differential expression of all genes between KO and WT starvation groups.**

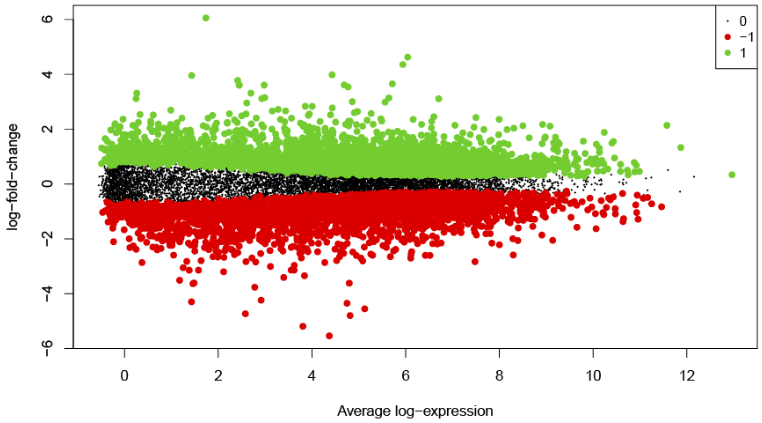

***Figure 7.* MDB plot showing differential expression of all genes between KO and WT refeed groups.**
